# A Review of Abrupt Permafrost Thaw: Definitions, Usage, and a Proposed Conceptual Framework

**DOI:** 10.1007/s40641-025-00204-3

**Published:** 2025-07-24

**Authors:** Hailey Webb, Matthias Fuchs, Benjamin W. Abbott, Thomas A. Douglas, Clayton D. Elder, Jessica Gilman Ernakovich, Eugenie S. Euskirchen, Mathias Göckede, Guido Grosse, Gustaf Hugelius, Miriam C. Jones, Charles Koven, Heather Kropp, Emma Lathrop, WenWen Li, Michael M. Loranty, Susan M. Natali, David Olefeldt, Christina Schädel, Edward A. G. Schuur, Oliver Sonnentag, Jens Strauss, Anna-Maria Virkkala, Merritt R. Turetsky

**Affiliations:** 1 Renewable and Sustainable Energy Institute, University of Colorado Boulder, Boulder, CO USA; 2 Department of Ecology and Evolutionary Biology, University of Colorado Boulder, Boulder, CO USA; 3 Department of Plant & Wildlife Sciences, Brigham Young University, Provo, UT USA; 4 U.S. Army Cold Regions Research and Engineering Laboratory, Fort Wainwright, AK 99703 USA; 5 Jet Propulsion Laboratory, California Institute of Technology, Pasadena, CA USA; 6 Earth Sciences Division, NASA Ames Research Center, Moffett Field, CA USA; 7 Center for Soil Biogeochemistry and Microbial Ecology, University of New Hampshire, Durham, NH USA; 8 Department of Natural Resources and the Environment, University of New Hampshire, Durham, NH USA; 9 Institute of Arctic Biology, University of Alaska Fairbanks, Fairbanks, AK 99775 USA; 10 Max Planck Institute for Biogeochemistry, Jena, Germany; 11 Alfred Wegener Institute Helmholtz Centre for Polar and Marine Research, Permafrost Research Section, 14473 Potsdam, Germany; 12 University of Potsdam, Institute of Geoscience, 14476 Potsdam, Germany; 13 Department of Physical Geography and Bolin Centre for Climate Research, Stockholm University, Stockholm, Sweden; 14 Florence Bascom Geoscience Center, U.S. Geological Survey, Reston, VA 20192 USA; 15 Earth Climate & Ecosystems Sciences, Lawrence Berkeley National Lab, Berkeley, CA 94720 USA; 16 Environmental Studies Program, Hamilton College, Clinton, NY USA; 17 Center for Ecosystem Science and Society, Northern Arizona University, Flagstaff, AZ 86001 USA; 18 School of Geographical Sciences and Urban Planning, Arizona State University, Tempe, AZ 85287-5302 USA; 19 Department of Geography, Colgate University, Hamilton, NY USA; 20 Woodwell Climate Research Center, Falmouth, MA 02540 USA; 21 Department of Renewable Resources, University of Alberta, Edmonton, Canada; 22 Université de Montréal, Département de Géographie, Montréal, QC H3C 3J7 Canada

**Keywords:** Arctic, Boreal, Climate feedbacks, Permafrost tipping points

## Abstract

**Purpose of Review:**

We review how ‘abrupt thaw’ has been used in published studies, compare these definitions to abrupt processes in other Earth science disciplines, and provide a definitive framework for how abrupt thaw should be used in the context of permafrost science.

**Recent Findings:**

We address several aspects of permafrost systems necessary for abrupt thaw to occur and propose a framework for classifying permafrost processes as abrupt thaw in the future. Based on a literature review and our collective expertise, we propose that abrupt thaw refers to thaw processes that lead to a substantial persistent environmental change within a few decades. Abrupt thaw typically occurs in ice-rich permafrost but may be initiated in ice-poor permafrost by external factors such as hydrologic change (i.e., increased streamflow, soil moisture fluctuations, altered groundwater recharge) or wildfire.

**Summary:**

Permafrost thaw alters greenhouse gas emissions, soil and vegetation properties, and hydrologic flow, threatening infrastructure and the cultures and livelihoods of northern communities. The term ‘abrupt thaw’ has emerged in scientific discourse over the past two decades to differentiate processes that rapidly impact large depths of permafrost, such as thermokarst, from more gradual, top-down thaw processes that impact centimeters of near-surface permafrost over years to decades. However, there has been no formal definition for abrupt thaw and its use in the scientific literature has varied considerably. Our standardized definition of abrupt thaw offers a path forward to better understand drivers and patterns of abrupt thaw and its consequences for global greenhouse gas budgets, impacts to infrastructure and land-use, and Arctic policy- and decision-making.

**Supplementary Information:**

The online version contains supplementary material available at 10.1007/s40641-025-00204-3.

## Main

Permafrost, or perennially frozen ground, underlies approximately 15% of land in the Northern Hemisphere [1] and regulates many characteristics of northern ecosystems including vegetation, hydrology, carbon balance, and nutrient cycling [2]. Permafrost is increasingly vulnerable to thaw, responding to rapid rates of Arctic change including warming [3], changes in precipitation and snow depth [4, 5], a longer snow-free season [6, 7], more intense wildfire regimes [8, 9], and land cover change [10–14]. Permafrost thaw occurs when previously frozen ground thaws and ground ice melts, resulting in consequences ranging from local impacts on land stability and navigation, to regional impacts on hydrology [15] and weather [16], to broader environmental and societal consequences such as loss of soil carbon storage and release of greenhouse gasses and industrial contaminants [17–20]. Beyond broad, generalized predictions, studies are just beginning to understand how ongoing and future permafrost thaw is likely to impact ground stability and infrastructure at local to regional scales [21]. For example, one study suggests thaw could cause widespread damage to infrastructure including an estimated 120,000 buildings, 40,000 km of roads, and 9,500 km of pipelines, affecting approximately 3.6 million people by 2050 [22] and costing billions of dollars to maintain [23]. Through feedbacks to the global climate system, carbon emissions from thawing permafrost are expected to release anywhere from 55–232 Pg carbon dioxide equivalent to the atmosphere by 2100, amplifying anthropogenic climate change [24]. The lower end of the range reflects projections where human efforts to limit anthropogenic greenhouse gas emissions keep global warming below 2 °C, while the upper end represents projected Arctic emissions with no carbon mitigation policies.

Permafrost research generally focuses on two broad categories of permafrost thaw that have different impacts on Arctic ecosystems and the global climate [25]. First, gradual top-down thaw leads to increasing thickness of the active layer – the surface layer of vegetation, soil, and sediment that freezes and thaws annually. This type of permafrost thaw occurs relatively slowly as near-surface permafrost thaws, impacting centimeters to meters of frozen ground over years to decades [26]. Gradual, top-down thaw (hereafter referred to as ‘gradual thaw’) occurs across the entire permafrost region, and because it is ubiquitous, tends to be well-studied [27, 28]. For example, active layer thickening has been monitored for several decades at more than 200 sites as part of the Circumpolar Active Layer Monitoring (CALM) network spanning much of the Arctic permafrost region [27]. There can be subsidence of the ground surface associated with gradual thaw; however, this parameter is not as widely measured as active layer thickening and our understanding of this process is limited [29–31]. In contrast to gradual thaw, the term ‘abrupt thaw’ is increasingly used to refer to a more pronounced type of permafrost thaw largely driven by the melting of ground ice and subsequent ground surface collapse. This includes geomorphological processes such as thermal and physical erosion [32], but the definition of abrupt thaw varies between studies. Abrupt thaw is often associated with the thawing of ice-rich (i.e., > 20% ice content) [33] permafrost and is suggested to rapidly (i.e., within months to years) impact meters of permafrost both horizontally and vertically through surface subsidence and erosion. Abrupt thaw creates characteristic landforms such as thaw lakes and wetlands, retrogressive thaw slumps, active-layer detachments, and thermal erosional gullies [34–36]. Thermokarst (the process by which characteristic landforms result from the thawing of ice-rich permafrost) [26] and thermal erosion (the erosion of ice-rich permafrost by the combined thermal and mechanical action of moving water) [26] are often used synonymously with abrupt thaw, but this narrow definition excludes processes such as wildfire-induced thaw which some scientists might consider to be an abrupt thaw process. Nevertheless, the development of thermokarst landscapes is an important abrupt thaw process, and thermokarst landscapes are estimated to store approximately 330 Pg soil organic carbon within the upper 3 m, representing about 30% of the total soil carbon stored in the top 3 m of the entire permafrost region [33].

In addition to near-surface carbon, abrupt thaw often exposes deep, previously frozen carbon to decomposition and erosion, making an increasingly large soil carbon pool (~ 400 Pg stored in deep Yedoma and deltaic deposits) vulnerable to release to the atmosphere [37]. Despite the potential to impact only a limited area of the permafrost region (collectively ~ 20%) [33], empirical studies show that abrupt thaw can lead to hotspots of permafrost carbon release in these regions [38–42]. Only two out of eleven Earth system models in the Intergovernmental Panel on Climate Change’s (IPCC) 6th Assessment Report represent some form of permafrost carbon feedback to climate, and none yet include abrupt thaw processes [43]. This is because large-scale models currently lack the architecture required to simulate the complex, three-dimensional changes in hydrology, vegetation, and surface elevation changes that drive the formation and expansion of abrupt thaw. They also lack representation of the three-dimensional structure of soil carbon and ground ice distribution that governs carbon release and thaw dynamics. As a result, abrupt thaw carbon release has been assessed using independent approaches outside of Earth system model frameworks, though it is acknowledged as one of the unresolved processes within Earth system models [44]. For example, first order inventory model simulations suggest net cumulative abrupt thaw carbon emissions on the order of 80 ± 19 Pg carbon by 2300, accounting for approximately 40% of the mean net emissions attributed to gradual thaw [38]. However, in these simulations, carbon emissions from abrupt thaw are expected to have the same radiative forcing and global warming impacts as gradual thaw due to large methane emissions. Still, carbon emissions from abrupt thaw remain difficult to upscale or model due to uncertainties in the current and future spatial extent of abrupt thaw, the diversity of thaw processes involved, their variability in carbon dioxide versus methane emissions, and the duration over which these processes release significant amounts of carbon. Due to their increasing occurrence, dramatic alteration of the landscape, and potential strong feedbacks to global climate, there is a need to more thoroughly study abrupt thaw processes. This first requires adequately defining and contextualizing abrupt thaw relative to other permafrost-specific geomorphic processes and general landforming and geological processes in the Earth system. In this paper, we review the varied historical use of the term abrupt thaw and build a framework for how it should be used consistently in the future.

## ‘Abrupt Thaw’ in the Literature

**Fig. 1:**
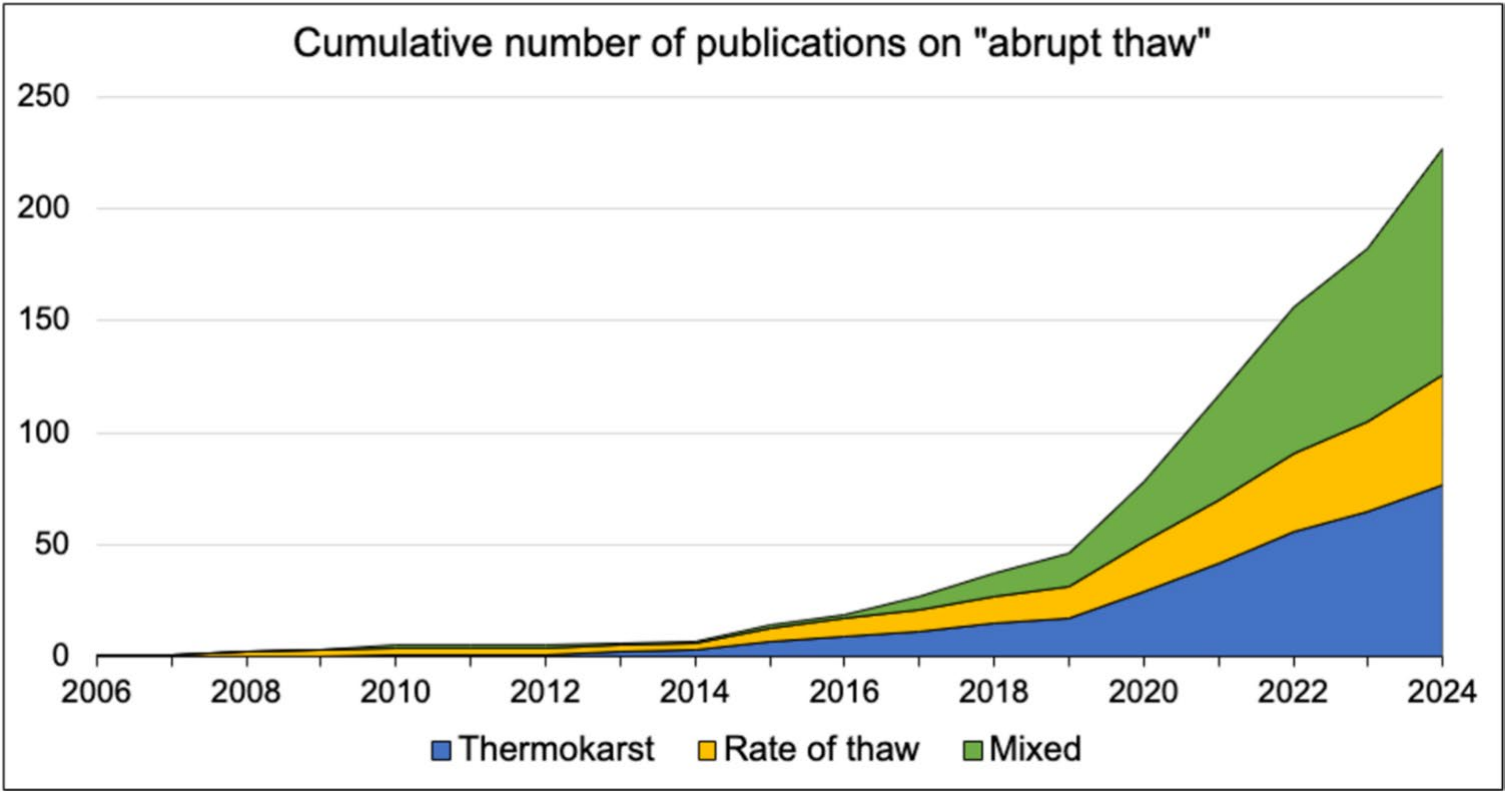
A plot of the cumulative number of publications per year using the term ‘abrupt thaw’ to describe either the occurrence of thermokarst/thermal erosion, a rapid temporal rate of thaw, or a mix of these categories

We conducted a literature search of peer-reviewed studies in journal articles, reports, and textbooks to explore the origin and evolution of the term ‘abrupt permafrost thaw’ (Supplementary Table 1). We used Web of Science and Google Scholar to identify studies that mention ‘abrupt thaw’ in the context of permafrost and either explicitly or implicitly define the term. Our search terms resulted in a review of 226 studies. Abrupt thaw was often used to describe thermokarst, thaw slumps, thermal erosional processes, or a rapid rate of thaw (days to years). However, definitions of abrupt thaw varied considerably among articles and were broad in scope. We found the usage of abrupt thaw can be grouped into three categories: formation of thermokarst and thermal erosion features, a rapid rate of thaw, or a combination of both thermokarst development and a rapid rate of thaw (Fig. 1).

Jorgenson et al. [32] initially described an abrupt increase in ice-rich permafrost degradation, resulting in thermokarst depression formation beyond normal rates of landscape change. The authors never specifically mentioned ‘abrupt thaw’ and instead used abrupt to describe a rapid change in permafrost conditions. Schuur et al. [45] also described abrupt thaw processes, stating, “Although permafrost thawing can occur gradually as the thickness of the active layer increases, it can also occur more abruptly through development of thermokarst (ground surface subsidence caused by thaw of ice-rich permafrost) and erosion.” The first use of ‘abrupt thaw’ was by Schuur & Abbott [46] who defined it as processes that “can cause ice wedges to melt and the ground surface to collapse, accelerating the thaw of frozen ground.” Most papers over the next few years continued to use abrupt thaw as synonymous with thermokarst or thermal erosion or a rate of thaw. Grosse et al. [36] described how rapid (pulse) disturbances are more associated with abruptly thawing permafrost and can be triggered by gradual (press) disturbances such as top-down permafrost thaw. Schneider von Deimling et al. [47] defined abrupt thaw as “thaw on a decadal scale” and included processes such as wildfire-induced thaw and coastal erosion. This was the first study to include processes other than thermokarst within the scope of abrupt thaw. They also noted that abrupt thaw must occur on sub-decadal time scales, using rate of thaw as the defining characteristic.

In 2016 and subsequent years, the use of abrupt thaw in the literature diversified, often defining abrupt thaw as a process that is both rapid and causes a pronounced physical change on the landscape without specifying the features that fall within those categories (Supplementary Table 1). Around the same time, a strong emphasis on ice-rich permafrost degradation emerged, stating that permafrost must be ice-rich for abrupt thaw to occur. The argument is that when ground ice melts, it causes the land surface to subside and amplifies rates of permafrost thaw through self-reinforcing feedbacks. Turetsky et al. [38] defined abrupt thaw as processes such as thermokarst that can affect meters of permafrost soil in periods of days to years [38]. This study was unique in its definition because it referenced the amount of thaw (in meters of impacted ground) within a given time frame for thaw processes to be considered abrupt.

Abrupt thaw is a useful term for communicating a broad range of geomorphological processes in a generalized, simple framework. However, our literature review reveals that abrupt thaw is used inconsistently. Definitions range from using the term synonymously with thermokarst to broadly encompassing a wide variety of processes (wildfire-induced thaw, coastal erosion, thermal erosion, ecosystem collapse) and timescales (days, weeks, months, years, decades). Most studies do not explicitly define abrupt thaw but instead give examples of a type of abrupt thaw such as thermokarst formation or thermal erosion, leaving room for other processes to be included in this definition without explicitly naming them.

## ‘Abrupt Change’ in Other Disciplines

It may be illuminating to frame abrupt thaw beyond the foundational fields, processes, and scales that define permafrost systems and their importance to society and climate. Here we draw on other disciplines and their definitions of abrupt change to identify key concepts that we think are useful to our goal of reaching a standardized definition of abrupt permafrost thaw.

Abrupt ecological change is defined as substantial changes in ecosystem states that occur in short periods of time relative to typical rates of change in the system or are rapid relative to their drivers [48]. Disturbances, such as wildfire or permafrost thaw, may impact ecosystems through changes in frequency or return interval, size, intensity, and severity of change [36, 49–55] (see Table 1). Abrupt ecological change is often used to describe disturbances that result in ecosystem state changes and may or may not be associated with thresholds or tipping points (see Table 1). Finally, abrupt ecological changes often result from interacting drivers. For example, in the context of permafrost systems, climate warming interacting with severe burning can trigger abrupt permafrost thaw.

**Table 1:**
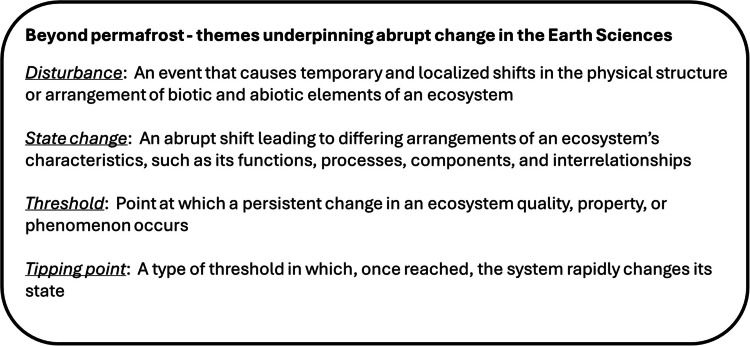
Conceptual box defining terms related to abrupt change in the Earth Sciences

Within the climate and geoscience fields, abrupt change has been defined by a rate of change (“a large change within less than 30 years” [56] and “a transition in the climate system whose duration is fast relative to the duration of the preceding or subsequent state” [57]). Thirty years is a relevant time frame because changes that occur in less than 30 years may have too much noise (i.e., influences of short-term fluctuations like weather and temporary ecosystem disturbances) while change occurring over a 30 + year period may not be relevant for effective policy making. Abrupt change has also been defined as a rapid initiation of a state change (“when the climate system is forced to cross some threshold, triggering a transition to a new state at a rate determined by the climate system itself and faster than the cause” [58]); a change involving a tipping point (abrupt changes that pass a critical threshold, or tipping point, after which cascading impacts on ecosystems may follow [59]); and the severity or magnitude of change (abrupt change has been defined by the magnitude of impact regardless of whether those changes occurred on time scales of centuries or 10,000 + years [60]).

In their review of abrupt climate and ecological change, Botta et al. [61] proposed that definitions of abrupt change should consider the frequency/timing, severity/magnitude, as well as the persistence of change. They propose the following definitions:

- Gradual change—change provoked by direct linear forcing.
- Rapid change—a large-scale change in the climate system that takes place over a few decades or less, persists (or is anticipated to persist) for at least a few decades and causes substantial disruptions in human and natural systems.
- Abrupt change—change that causes a system to cross a tipping point and switch to a new state.

These frameworks are helpful for merging key concepts found across the ecological, climate, and geological disciplines reviewed here. We apply these concepts to the permafrost system to articulate a definition of abrupt thaw that can be utilized in a more consistent way across permafrost studies. We conclude that a definition of abrupt thaw should include: (1) thaw that occurs more rapidly than typical permafrost degradation (i.e., gradual top-down thaw), and (2) thaw that leads to substantial alteration of the structure, integrity/stability, or function of permafrost systems that persists longer than those caused by gradual thaw.

## Key Questions to Consider when Seeking a Definition of Abrupt Permafrost Thaw

**Fig. 2:**
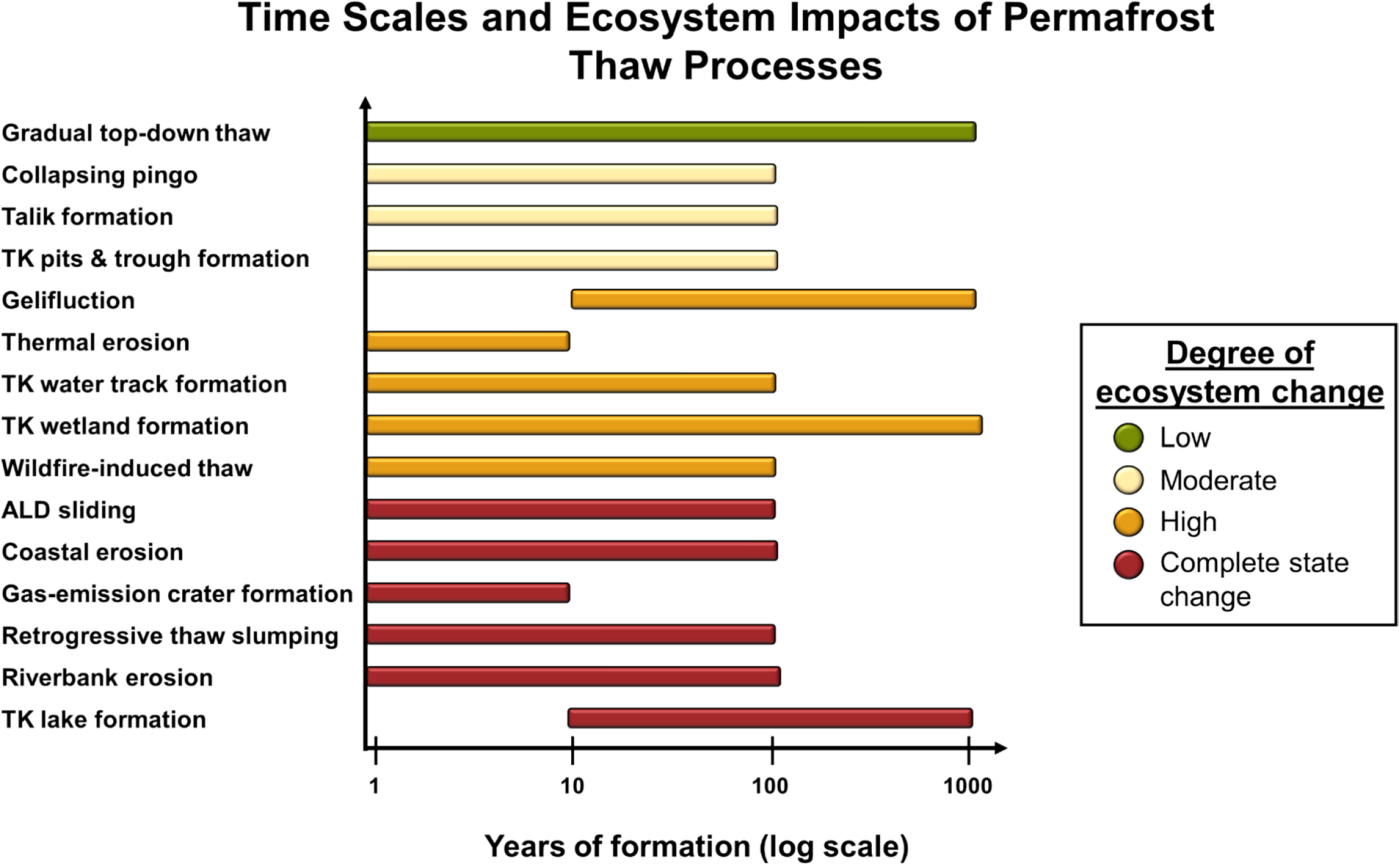
Permafrost thaw processes at the landform scale showing the time it takes for each process to form and the degree of ecosystem change. Definitions of the degree of ecosystem change categories were adapted from Turner et al. (2020) [48] and are detailed in Supplementary Table 2. Abbreviations: Thermokarst (TK) and Active layer detachment (ALD)

**Fig. 3:**
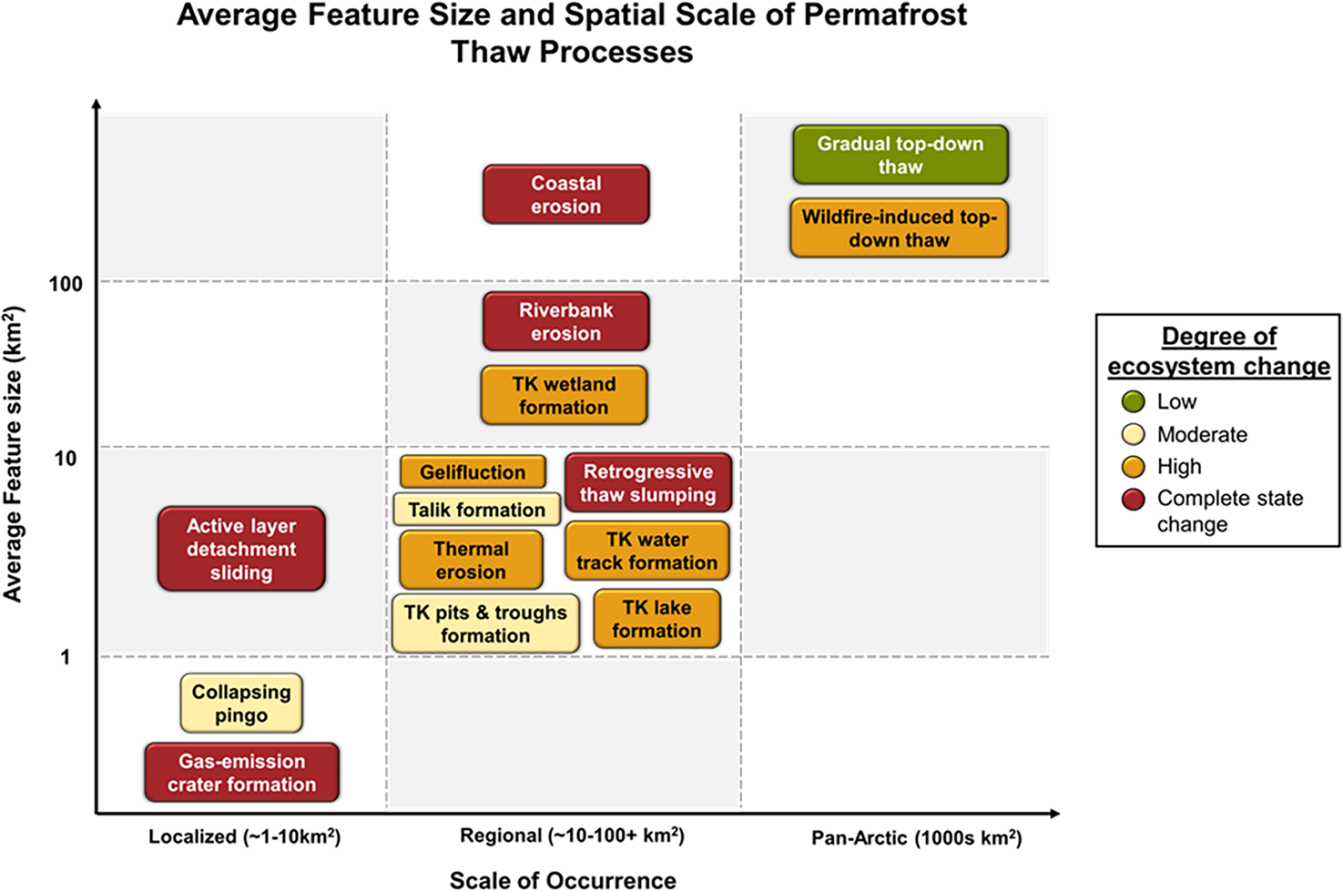
The average feature size and scale of occurrence for permafrost thaw processes. The scale of occurrence reflects the maximum extent of the permafrost region likely to be affected by each process. Feature sizes are based on average estimates and individual instances may occur outside the ranges shown. The degree of ecosystem change is represented by the color of each word bubble. Abbreviations: Thermokarst (TK)

We developed conceptual diagrams to visualize the formation time, degree/magnitude of ecosystem change, average feature size, and scale of impacts for common permafrost thaw processes (Fig. 2 and 3). We used mean literature values from 44 studies and our expert assessment to place a variety of permafrost thaw processes into these two-dimensional spaces (Supplementary Table 3). In some cases, disturbances such as wildfire or human activity (i.e., seismic lines or other linear features [62, 63]) remove the surface organic layers that insulate permafrost and amplify both thaw rates and ecosystem change. Here we consider the cumulative impacts of these changes as there is insufficient information to examine processes individually. We acknowledge that across all types of permafrost thaw processes, the rate of permafrost change and the magnitude of ecosystem impacts vary widely in relation to ice content, vegetation type, and other key driving factors. We use the information from Fig. 2 and 3 to explore several conditions of the permafrost system and ask how they are related to the potential for abrupt thaw.

### How Rapidly Does Permafrost need to Thaw for it to be Considered Abrupt?

Thaw processes can be classified as abrupt thaw when they occur rapidly (less than 30 years) and cause large ecosystem changes or complete state change. Conversely, processes cannot be considered abrupt thaw when they occur on the same time scales as gradual top-down thaw (30 + years to centuries) and have low to moderate ecosystem impacts. Abrupt thaw processes must be initiated within that 30-year time interval, however, it may take more than 30 years for a feature to fully develop. Using thermokarst lake formation as an example, melting ice wedges may cause meters of ground subsidence over the course of a decade, but the lake can continue to deepen and expand gradually over the course of a century. This is still considered abrupt thaw because of the rapid initiation of the feature. The ambiguity lies with thaw processes that occur rapidly but do not have large ecosystem impacts. For example, talik formation (perennially thawed regions of ground found in a permafrost environment) [64] can happen rapidly but this individual process may not cause significant changes to the ecosystem. The primary distinction between rapid and abrupt thaw is that rapid thaw refers solely to the rate of permafrost thaw, whereas abrupt thaw also entails substantial and persistent changes to ecosystem structure or function. For instance, wildfires typically lead to rapid permafrost thaw due to combustion of the insulating organic soil layer and albedo reductions [65]. However, wildfire-induced permafrost thaw can only be classified as abrupt if it is accompanied by ground subsidence, altered local hydrology, or some other change to ecosystem identity and function. We conclude that the definition of abrupt thaw should not be based solely on the rate of thaw-induced change but should also include the processes causing long-lasting and substantial impacts to the ecosystem.

### Does Abrupt Thaw Always Occur in Ice-Rich Permafrost?

Ground ice is a key factor contributing to abrupt thaw processes, but not required. Ground ice originates from rain, spring snowmelt, buried glacial ice, snow patches, or freshwater pond ice. However, ground ice is extremely heterogeneous over many scales and may range from pore ice, a barely visible cement holding silt and sand grains together, to centimeters-thick segregation ice common at the base of the active layer, to large massive ice bodies formed by different processes in the past. Massive ice wedges can be tens of meters tall and the ubiquitous presence of readily visible “patterned ground” indicates their presence over much of the permafrost domain [66]. Buried glacial ice consists of sometimes tens of meters thick ice beds extending over kilometers in regions that were formerly glaciated [67]. The presence and colocation of these ice features, their bulk density, and their formation histories are difficult to quantify at large scales. Regardless of its formation history, the melting of excess ground ice (massive ice bodies or segregated ice exceeding the normal pore space volume of a sediment) causes rapid geomorphologic changes. The phase change from ice to water decreases volume by 9%, creating voids and channels in the surface. Surface subsidence leads to changes in surface elevation at a variety of scales. The degree of soil or sediment subsidence post-thaw is directly correlated to the ground ice content of permafrost. In many instances, once surface subsidence has initiated, multiple positive feedback mechanisms may result in an acceleration of thaw and subsidence, eventually resulting in abrupt landform change. These positive feedbacks increase heat influx into the ground, including water pooling and snow accumulation in subsided areas and enhanced erosion. In contrast, permafrost with extensive ground ice can experience longer thaw duration due to the greater amount of energy necessary for the phase change from ice to liquid water.

While the peer-reviewed literature often defines abrupt thaw as only occurring in ice-rich permafrost, we identified a number of processes that occur rapidly with significant ecosystem impacts that do not necessarily occur only in ice-rich permafrost, such as wildfire-induced top-down thaw, riverbank erosion, and coastal erosion (Fig. 2). From this, we conclude that while ground ice is an important factor contributing to abrupt thaw in some areas, abrupt thaw does not occur solely in ice-rich permafrost and thus the definition of abrupt thaw should not be based on the presence of ground ice.

### How do Thaw Mechanisms, the Scale of Occurrence, and Ecological Impact Vary for Abrupt Thaw Processes?

Multiple thaw processes can occur in an ecosystem at the same time, and some processes can trigger or amplify others. For example, talik formation can eventually lead to the formation of thermokarst features or vice versa [68, 69]. Additionally, rapid active layer thickening caused by severe wildfire can trigger active layer detachment slides in sloped areas. Many of these thaw processes are linked and the dominant force of thaw in these systems may change over time with shifts in ecosystem function and identity. None of this complexity is captured in large-scale models, as most modeling frameworks lack the capability to represent any form of abrupt thaw.

Some thaw processes are more common and impact larger portions of the permafrost region while others are less frequent or extensive. For example, in boreal peatlands and lowlands, wetland thermokarst features can occupy > 60% of the landscape [33], causing significant disruption to local infrastructure and ecosystems and releasing substantial amounts of greenhouse gases, particularly methane [39, 40]. On the other hand, gas-emission craters are a very dramatic and extreme version of abrupt change in permafrost systems. They are formed by an explosion that leaves behind a massive crater measuring dozens of meters wide and deep [70]. There is uncertainty about whether the craters are formed as a result of temperature or hydrologically-driven permafrost thaw or mechanical permafrost degradation from belowground gas buildup and pressure [71, 72]. Although these events receive lots of media attention due to their impressive size and rapid appearance on the landscape [73–75], these craters are isolated and have only been observed in the northern part of the West-Siberian Plain on or near the Yamal Peninsula [70, 76]. Even though the impact of gas-emission craters on the local terrain is substantial, their socioecological consequences on ecosystem- to climate-system scales are more limited. As such, the diversity of abrupt thaw types and both scope and scale of their consequences may necessitate different abrupt thaw classification schemes for various purposes. Local infrastructure planning for example may need to place priority on understanding different abrupt thaw features and their impact on local terrain land use change.

### How do Abrupt Thaw Processes Differ in their Contribution to the Permafrost Carbon Feedback?

Thaw processes can vary in their contribution to greenhouse gas emissions, depending on the volume of thaw-affected permafrost as well as the biogeochemical and hydrological setting resulting from thaw. In general, lowland thaw features tend to be wetter and emit more methane than upland thaw features [38]. Within lowland thaw areas, thermokarst lakes can vary depending on their surrounding permafrost characteristics and as a result can be high or low emitting sources for biogenic methane [77]. Some lakes develop deep taliks that facilitate tapping into deeper geologic gas sources, allowing natural gas seepage from sedimentary basins to occur [78]. Thus, some thermokarst lakes emit significantly more methane than others, highlighting the variability in greenhouse gas emissions within a single type of abrupt thaw feature. This variability within features can substantially affect their contribution to the permafrost carbon feedback and broader climate change. Accounting for this variation requires knowledge not only of abrupt thaw features but also the role of biogeochemical cycling, permafrost history, geological substrates, hydrology, and vegetation in regulating greenhouse gas emissions and lateral carbon release [79, 80]. The current and potential emissions associated with each abrupt thaw process shown in Fig. 2 remain largely unknown, but some estimates have been made based on limited data from collapse-scar wetlands, thermokarst lakes, and hillside erosion [38, 81–84]. While regional to pan-Arctic scale processes are expected to have a greater impact on carbon emissions than localized processes, there is a lack of supporting data. Improving our understanding of these emissions should be a priority for future research.

Abrupt thaw landforms also vary in their trajectory of recovery following permafrost thaw, including the rate of ecosystem stabilization and possible reformation of permafrost. Thermokarst may trigger large carbon releases in the short-term but eventually transition to an ecosystem state that accumulates soil carbon, often facilitated by lake drainage and fen/bog vegetation expansion [85–87]. Wildfire can increase ground thermal conductivity and reduce surface albedo causing rapid active layer thickening [88] and long-term permafrost thaw. Some permafrost is resilient to this type of disturbance and can stabilize and even re-aggrade within several decades [65, 89]. While gradual thaw mostly causes linear changes to permafrost systems over time, the long-term impacts of abrupt thaw are difficult to predict because of complex successional trajectories. In landforms undergoing abrupt thaw, the forcing (typically rising air and/or soil temperatures) often triggers nonlinear effects on the rate of permafrost thaw. A prime example of this is ice-rich permafrost thaw leading to thermokarst formation. Gradually rising air temperatures thaw the upper layers of permafrost by a few centimeters each year, but when the thaw front reaches ice, liquid water can trigger rapid thaw causing meters of subsidence in one season or year in its most actively thawing stage. Once most of the ice has finished melting, thaw rates will often decrease and stabilize as the thermokarst feature begins to enter a new steady state. However, changes in vegetation caused by wetting and subsidence can create positive feedbacks to additional surface warming that can accelerate the rate of thermokarst development [90]. Such non-linear responses differentiate abrupt from gradual permafrost thaw processes but also highlight the difficulties of simulating abrupt thaw in Earth system models.

## Modeling Abrupt Thaw

Modeling abrupt thaw is particularly challenging due to several limitations. Because of the high computational demands of simulating fine-scale landscape heterogeneity, most land components of Earth system models (ESMs) operate at coarse resolutions (i.e., ≥ 1 km) which are too large to identify small-scale phenomena such as individual thermokarst features that occur over meters to 10s of meters. Incorporating abrupt thaw into models is further complicated by a limited understanding of: current abrupt thaw feature distribution and rate of growth/stabilization; where, when, and how quickly future abrupt thaw features will form at the pan-Arctic scale; greenhouse gas emissions from abrupt thaw features (due to a lack of data and potential research bias towards high-emitting abrupt thaw features); and the substantial variability in emissions even within the same feature type. It is particularly difficult to simulate the dynamic interactions between the ground surface, water table, and frost table that cause shifts in ground subsidence and hydrology during abrupt thaw processes, though fine-scale efforts are underway [91]. These surfaces shift in complex, three-dimensional patterns which differ from the more one-dimensional increase in active layer thickness typically associated with gradual thaw. Wildfire-induced abrupt thaw may be more tractable than thermokarst or other fine-scale processes due to the larger spatial scale of wildfire and efforts to resolve heterogeneity in wildfire and other disturbance processes in models. These disturbances, often driven by the redistribution of water linked to geomorphological changes, not only affect the immediate area but also impact the broader landscape, further complicating large-scale assessments of abrupt thaw’s impacts. Another limitation is the lack of fine-scale ground ice maps. Ground ice plays a crucial role in driving many abrupt thaw processes, but mapping its extent at fine spatial scales remains a challenge because it is a subsurface feature that cannot be directly observed using satellite imagery. However, there have been attempts to map small scale abrupt thaw permafrost features with high-resolution satellite imagery [92–95].

Long-term hydrologic uncertainty adds another layer of complexity: it remains unclear if and when thermokarst lakes will drain, resulting in major implications for greenhouse gas emissions [40]. Methane is more likely to be produced under wet conditions, resulting in more potent short-term greenhouse gas effects, while drier conditions are associated with more carbon dioxide release [37, 96]. Finally, the age and formation history of abrupt thaw features may influence their emissions [97]. Some present-day thermokarst lakes are thought to have repeatedly formed and drained since the end of the Pleistocene [98], suggesting their soil carbon has already undergone some level of microbial decomposition one or more times [99]. In contrast, newly formed thermokarst lakes may expose previously frozen, carbon-rich soils for the first time, potentially leading to higher emissions. These uncertainties highlight the need for a better understanding of how to define abrupt thaw so that we can prioritize more research into this type of thaw and improve emissions projections from permafrost soils.

New approaches using artificial intelligence (AI) offer a promising path for improving abrupt thaw feature mapping and modeling, starting with targeted pilot studies that leverage data-driven methods to capture fine-scale thaw dynamics from multi-source data (i.e., ground observations and drone or satellite imagery) with greater computational efficiency [100, 101]. These models can uncover emergent patterns and relationships that are difficult to represent using traditional physics-based approaches alone. Insights gained from such studies can then inform and refine our physical understanding of thaw processes. By integrating this knowledge into ESMs – either through hybrid modeling or by constraining AI models with known physical processes – we can improve the representation of abrupt thaw in large-scale climate simulations.

## A Framework for Classifying Abrupt Thaw

**Fig. 4:**
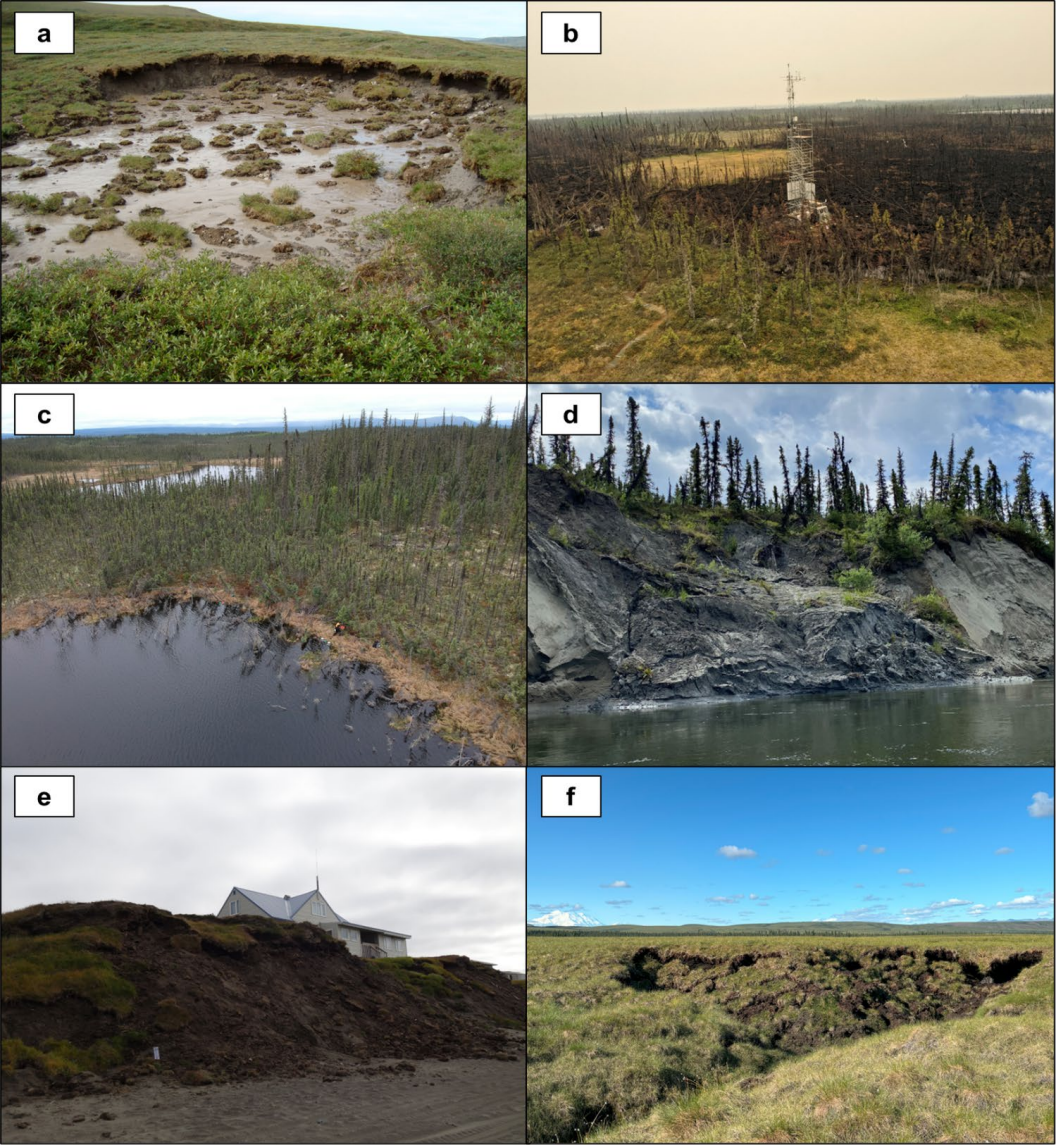
Photographs of common abrupt thaw landforms: **a**) retrogressive thaw slump near Toolik Field Station, Alaska, USA; **b**) wildfire-induced thaw at Scotty Creek Landscape, a flux tower of the AmeriFlux network (AmeriFlux ID: CA-SCC) near Fort Simpson, NT, Canada; **c**) thermokarst lake near Wrigley, NT, Canada; **d**) riverbank erosion along the Gulkana River, Alaska, USA; **e**) coastal erosion near Utqiaġvik, Alaska, USA; **f**) thermal erosion gully in Denali National Park, Alaska, USA

**Fig. 5:**
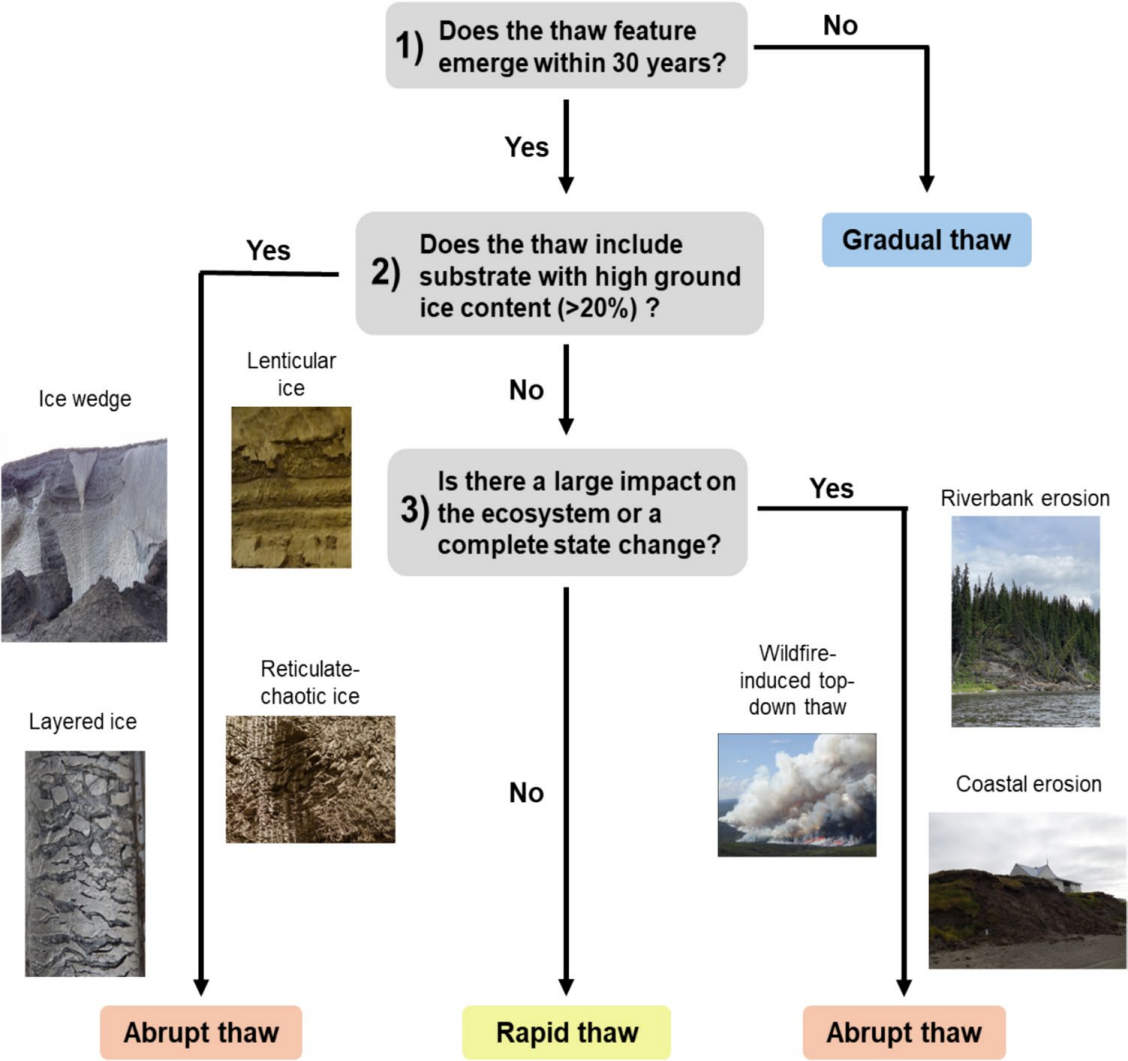
Decision tree outlining the method of classification for abrupt, rapid, and gradual thaw processes/features at the landform scale. Note that this decision tree refers to permafrost thaw and not physical degradation of permafrost such as mining, drilling, or infrastructure development

Our literature review and analyses considered three defining characteristics of permafrost thaw processes: 1) the mean rates of thaw, 2) the degree of ecosystem change, and 3) whether or not the process requires high ground ice content. We conclude that abrupt permafrost thaw includes processes by which permafrost thaws within a season to a few decades and causes substantial and sustained geomorphological, hydrogeological, or ecological changes to the system, specifically at the landform scale (Fig. 4). This process can happen in ice-rich permafrost where internal feedbacks from melting ground ice or subsidence-driven changes in vegetation accelerate thaw. However, abrupt thaw can also occur when an external factor such as wildfire, changing hydrology, or gas buildup causes permafrost to thaw or deform faster than it would by air or soil temperature changes alone. This can occur in systems underlain both by ice-rich and ice-poor permafrost. Using this definition at the landform scale, we outline a simple decision tree to determine whether or not a thaw process or feature should be classified as abrupt thaw (Fig. 5).

In landforms with high (> 20%) ground ice content, thaw typically will be abrupt due to hydrologic change and significant volume loss. In some instances, abrupt thaw can be preceded by many years of gradual thaw processes until the thaw front reaches the ice-rich substrate. Abrupt thaw in ice-poor landforms requires an external factor to the system instigating thaw beyond the typical rates of permafrost thaw occurring with rising air or soil temperatures alone. Examples include: rapid top-down thaw caused by an extreme wildfire resulting in active layer deepening at rates much faster than would have been expected without the influence of fire; rapid coastal erosion caused by rising sea levels; and riverbank erosion caused by increased glacial runoff and higher water levels.

## Continued Use of the Term “abrupt thaw”

We show that abrupt thaw has been embedded in the literature for over a decade. Abrupt thaw was initially used to describe rapid permafrost thaw and over time developed into a multi-faceted term to describe complex geomorphological and environmental conditions that may cause permafrost to thaw faster than anticipated by an increase in air or soil temperature alone. Definitions vary across publications and the term is used inconsistently. Nevertheless, the concept of ‘abrupt thaw’ is helpful for categorizing thaw processes distinct from gradual top-down thaw. This concept also encapsulates the extensive, technical permafrost terminology in a way that will be more easily understood by the public, media, and may also offer insight into how these permafrost processes may be generalized for inclusion in large scale models [38]. Discontinuing the use of the term abrupt thaw would be a disservice due to its widespread use in the literature and its educational power in science communication efforts. However, abrupt thaw should not be used as synonymous with thermokarst/thermal erosion. In most cases, using a more specific reference to thermokarst or thermal erosion is more appropriate and lessens ambiguity because precise definitions of these terms have already been resolved. These specific terms are beneficial when discussing geomorphologic processes and the development of particular landforms. We suggest that abrupt thaw should be used when communicating to generalized audiences, describing multiple types of abrupt thaw processes, or dealing with general concepts of abrupt permafrost change.

We recommend that continued use of the term ‘abrupt thaw’ follow the classification outlined in Fig. 5. Adopting this formalized method for abrupt thaw classification will lessen ambiguity surrounding the term and improve future research on the causes and consequences of this broad suite of permafrost changes. Most efforts to model greenhouse gas contributions to the climate system from thawing permafrost have only considered gradual top-down thaw [43]. However, several studies focusing specifically on abrupt thaw have demonstrated that these processes serve as hotspots for greenhouse gas emissions [38–41, 102, 103], and that we will underestimate the permafrost carbon feedback to climate without considering carbon emissions from abrupt thaw features.

Beyond climate feedbacks, abrupt thaw is destabilizing soils and coastlines, forming areas of flooding and inundation in some regions and leading to catastrophic erosion and lake drainage in others. Because abrupt thaw invokes significant changes to landscapes and the ecosystem services that many northern Indigenous and non-Indigenous communities rely upon, it is imperative to incorporate abrupt thaw into current and future land planning and management schemes across the Arctic. Our study provides clarity about what constitutes abrupt thaw and how this term should be used consistently in the future, which we believe will be imperative for both scientific research as well as meaningful policy related to Arctic change.

## Supplementary Information

Below is the link to the electronic supplementary material.

Supplementary file1 (DOCX 288 KB)

## Data Availability

No datasets were generated or analysed during the current study.

## References

[1] Obu J. How much of the earth’s surface is underlain by permafrost? J Geophys Res Earth Surf. 2021;126:e2021JF006123. 10.1029/2021JF006123.

[2] Schuur EAG, Mack MC. Ecological response to permafrost thaw and consequences for local and global ecosystem services. Annu Rev Ecol Evol Syst. 2018;49:279–301. 10.1146/annurev-ecolsys-121415-032349.

[3] Rantanen M, Karpechko AYu, Lipponen A, Nordling K, Hyvärinen O, Ruosteenoja K, Vihma T, Laaksonen A. The arctic has warmed nearly four times faster than the globe since 1979. Commun Earth Environ. 2022;3:168. 10.1038/s43247-022-00498-3.

[4] Douglas TA, Turetsky MR, Koven CD. Increased rainfall stimulates permafrost thaw across a variety of interior Alaskan boreal ecosystems. Npj Clim Atmospheric Sci. 2020;3:28. 10.1038/s41612-020-0130-4.

[5] Stieglitz M, Déry SJ, Romanovsky VE, Osterkamp TE. The role of snow cover in the warming of arctic permafrost. Geophys Res Lett. 2003;30:2003GL017337. 10.1029/2003GL017337.

[6] Kim Y, Kimball JS, Zhang K, McDonald KC. Satellite detection of increasing Northern Hemisphere non-frozen seasons from 1979 to 2008: implications for regional vegetation growth. Remote Sens Environ. 2012;121:472–87. 10.1016/j.rse.2012.02.014.

[7] Callaghan TV, Johansson M, Brown RD, Groisman PYA, Labba N, Radionov V, Barry RG, Bulygina ON, Essery RLH, Frolov DM, Golubev VN, Grenfell TC, Petrushina MN, Razuvaev VN, Robinson DA, Romanov P, Shindell D, Shmakin AB, Sokratov SA, Warren S, Yang D. The changing face of arctic snow cover: a synthesis of observed and projected changes. Ambio. 2011;40:17–31. 10.1007/s13280-011-0212-y.

[8] Bintanja R. The impact of Arctic warming on increased rainfall. Sci Rep. 2018;8:16001. 10.1038/s41598-018-34450-3. 30375466 10.1038/s41598-018-34450-3 PMC6207739

[9] Descals A, Gaveau DLA, Verger A, Sheil D, Naito D, Peñuelas J. Unprecedented fire activity above the arctic circle linked to rising temperatures. Science. 2022;378:532–7. 10.1126/science.abn9768. 36378957 10.1126/science.abn9768

[10] Jorgenson MT, Grosse G. Remote sensing of landscape change in permafrost regions. Permafr Periglac Process. 2016;27:324–38. 10.1002/ppp.1914.

[11] Carpino O, Haynes K, Connon R, Craig J, Devoie É, Quinton W. Long-term climate-influenced land cover change in discontinuous permafrost peatland complexes. Hydrol Earth Syst Sci. 2021;25:3301–17. 10.5194/hess-25-3301-2021.

[12] Foster AC, Wang JA, Frost GV, Davidson SJ, Hoy E, Turner KW, Sonnentag O, Epstein H, Berner LT, Armstrong AH, Kang M, Rogers BM, Campbell E, Miner KR, Orndahl KM, Bourgeau-Chavez LL, Lutz DA, French N, Chen D, Du J, Shestakova TA, Shuman JK, Tape K, Virkkala A-M, Potter C, Goetz S. Disturbances in North American boreal forest and arctic tundra: impacts, interactions, and responses. Environ Res Lett. 2022;17:113001. 10.1088/1748-9326/ac98d7.

[13] Klotz LA, Sonnentag O, Wang Z, Wang JA, Kang M. Oil and natural gas wells across the NASA ABoVE domain: fugitive methane emissions and broader environmental impacts. Environ Res Lett. 2023;18:035008. 10.1088/1748-9326/acbe52.

[14] Stuenzi SM, Boike J, Gädeke A, Herzschuh U, Kruse S, Pestryakova LA, Westermann S, Langer M. Sensitivity of ecosystem-protected permafrost under changing boreal forest structures. Environ Res Lett. 2021;16:084045. 10.1088/1748-9326/ac153d.

[15] Walvoord MA, Kurylyk BL. Hydrologic impacts of thawing permafrost—a review. Vadose Zone J. 2016;15:1–20. 10.2136/vzj2016.01.0010.

[16] Helbig M, Wischnewski K, Kljun N, Chasmer LE, Quinton WL, Detto M, Sonnentag O. Regional atmospheric cooling and wetting effect of permafrost thaw-induced boreal forest loss. Glob Change Biol. 2016;22:4048–66. 10.1111/gcb.13348. 10.1111/gcb.13348 27153776

[17] Schuur EAG, Vogel JG, Crummer KG, Lee H, Sickman JO, Osterkamp TE. The effect of permafrost thaw on old carbon release and net carbon exchange from tundra. Nature. 2009;459:556–9. 10.1038/nature08031. 19478781 10.1038/nature08031

[18] Christensen K. Thawing permafrost releases industrial contaminants into arctic communities. Environ Health Perspect. 2024;132:032001. 10.1289/EHP13998. 38536884 10.1289/EHP13998 PMC10971047

[19] Langer M, Von Deimling TS, Westermann S, Rolph R, Rutte R, Antonova S, Rachold V, Schultz M, Oehme A, Grosse G. Thawing permafrost poses environmental threat to thousands of sites with legacy industrial contamination. Nat Commun. 2023;14:1721. 10.1038/s41467-023-37276-4. 36977724 10.1038/s41467-023-37276-4 PMC10050325

[20] Plaza C, Pegoraro E, Bracho R, Celis G, Crummer KG, Hutchings JA, Hicks Pries CE, Mauritz M, Natali SM, Salmon VG, Schädel C, Webb EE, Schuur EAG. Direct observation of permafrost degradation and rapid soil carbon loss in tundra. Nat Geosci. 2019;12:627–31. 10.1038/s41561-019-0387-6.

[21] Fatolahzadeh Gheysari A, Maghoul P. A framework to assess permafrost thaw threat for land transportation infrastructure in northern Canada. Commun Earth Environ. 2024;5:167. 10.1038/s43247-024-01317-7.

[22] Hjort J, Karjalainen O, Aalto J, Westermann S, Romanovsky VE, Nelson FE, Etzelmüller B, Luoto M. Degrading permafrost puts Arctic infrastructure at risk by mid-century. Nat Commun. 2018;9:5147. 10.1038/s41467-018-07557-4. 30538247 10.1038/s41467-018-07557-4 PMC6289964

[23] Suter L, Streletskiy D, Shiklomanov N. Assessment of the cost of climate change impacts on critical infrastructure in the circumpolar Arctic. Polar Geogr. 2019;42:267–86. 10.1080/1088937X.2019.1686082.

[24] Schuur EAG, Abbott BW, Commane R, Ernakovich J, Euskirchen E, Hugelius G, Grosse G, Jones M, Koven C, Leshyk V, Lawrence D, Loranty MM, Mauritz M, Olefeldt D, Natali S, Rodenhizer H, Salmon V, Schädel C, Strauss J, Treat C, Turetsky M. Permafrost and climate change: carbon cycle feedbacks from the warming arctic. Annu Rev Environ Resour. 2022;47:343–71. 10.1146/annurev-environ-012220-011847.

[25] Nitzbon J, Schneider Von Deimling T, Aliyeva M, Chadburn SE, Grosse G, Laboor S, Lee H, Lohmann G, Steinert NJ, Stuenzi SM, Werner M, Westermann S, Langer M. No respite from permafrost-thaw impacts in the absence of a global tipping point. Nat Clim Change. 2024;14:573–85. 10.1038/s41558-024-02011-4.

[26] Subcommittee P. Glossary of permafrost and related ground-ice terms. Assoc Comm Geotech Res Natl Res Counc Can Ott. 1988;156:63–4.

[27] Brown J, Hinkel KM, Nelson FE. The circumpolar active layer monitoring (calm) program: research designs and initial results. Polar Geogr. 2000;24:166–258. 10.1080/10889370009377698.

[28] Hinkel KM, Nelson FE. Spatial and temporal patterns of active layer thickness at circumpolar active layer monitoring (CALM) sites in northern Alaska, 1995–2000. J Geophys Res Atmospheres. 2003;108:2001JD000927. 10.1029/2001JD000927.

[29] O’Neill HB, Smith SL, Burn CR, Duchesne C, Zhang Y. Widespread permafrost degradation and thaw subsidence in Northwest Canada. J Geophys Res Earth Surf. 2023;128:e2023JF007262. 10.1029/2023JF007262.

[30] Rodenhizer H, Ledman J, Mauritz M, Natali SM, Pegoraro E, Plaza C, Romano E, Schädel C, Taylor M, Schuur E. Carbon thaw rate doubles when accounting for subsidence in a permafrost warming experiment. J Geophys Res Biogeosciences. 2020;125:e2019JG005528. 10.1029/2019JG005528.

[31] Streletskiy DA, Maslakov A, Grosse G, Shiklomanov NI, Farquharson L, Zwieback S, Iwahana G, Bartsch A, Liu L, Strozzi T, Lee H, Debolskiy MV. Thawing permafrost is subsiding in the Northern Hemisphere—review and perspectives. Environ Res Lett. 2025;20:013006. 10.1088/1748-9326/ada2ff.

[32] Jorgenson MT, Shur YL, Pullman ER. Abrupt increase in permafrost degradation in arctic Alaska. Geophys Res Lett. 2006;33:L02503. 10.1029/2005GL024960.

[33] Olefeldt D, Goswami S, Grosse G, Hayes D, Hugelius G, Kuhry P, McGuire AD, Romanovsky VE, Sannel ABK, Schuur EAG, Turetsky MR. Circumpolar distribution and carbon storage of thermokarst landscapes. Nat Commun. 2016;7:13043. 10.1038/ncomms13043. 27725633 10.1038/ncomms13043 PMC5062615

[34] Jorgenson MT, Osterkamp TE. Response of boreal ecosystems to varying modes of permafrost degradation. Can J For Res. 2005;35:2100–11. 10.1139/x05-153.

[35] Jones BM, Amundson CL, Koch JC, Grosse G. Thermokarst and thaw-related landscape dynamics—an annotated bibliography with an emphasis on potential effects on habitat and wildlife: U.S. Geological Survey Open-File Report 2013-1161; 2013. p. 60. http://pubs.usgs.gov/of/2013/1161.

[36] Grosse G, Harden J, Turetsky M, McGuire AD, Camill P, Tarnocai C, Frolking S, Schuur EAG, Jorgenson T, Marchenko S, Romanovsky V, Wickland KP, French N, Waldrop M, Bourgeau-Chavez L, Striegl RG. Vulnerability of high-latitude soil organic carbon in North America to disturbance. J Geophys Res. 2011;116:G00K06. 10.1029/2010JG001507.

[37] Schuur EAG, McGuire AD, Schädel C, Grosse G, Harden JW, Hayes DJ, Hugelius G, Koven CD, Kuhry P, Lawrence DM, Natali SM, Olefeldt D, Romanovsky VE, Schaefer K, Turetsky MR, Treat CC, Vonk JE. Climate change and the permafrost carbon feedback. Nature. 2015;520:171–9. 10.1038/nature14338. 25855454 10.1038/nature14338

[38] Turetsky MR, Abbott BW, Jones MC, Anthony KW, Olefeldt D, Schuur EAG, Grosse G, Kuhry P, Hugelius G, Koven C, Lawrence DM, Gibson C, Sannel ABK, McGuire AD. Carbon release through abrupt permafrost thaw. Nat Geosci. 2020;13:138–43. 10.1038/s41561-019-0526-0.

[39] Walter Anthony KM, Lindgren P, Hanke P, Engram M, Anthony P, Daanen RP, Bondurant A, Liljedahl AK, Lenz J, Grosse G, Jones BM, Brosius L, James SR, Minsley BJ, Pastick NJ, Munk J, Chanton JP, Miller CE, Meyer FJ. Decadal-scale hotspot methane ebullition within lakes following abrupt permafrost thaw. Environ Res Lett. 2021;16:035010. 10.1088/1748-9326/abc848.

[40] Walter Anthony K, Schneider von Deimling T, Nitze I, Frolking S, Emond A, Daanen R, Anthony P, Lindgren P, Jones B, Grosse G. 21st-century modeled permafrost carbon emissions accelerated by abrupt thaw beneath lakes. Nat Commun. 2018;9:3262. 10.1038/s41467-018-05738-9. 30111815 10.1038/s41467-018-05738-9 PMC6093858

[41] Rodenhizer H, Belshe F, Celis G, Ledman J, Mauritz M, Goetz S, Sankey T, Schuur EAG. Abrupt permafrost thaw accelerates carbon dioxide and methane release at a tussock tundra site. Arct Antarct Alp Res. 2022;54:443–64. 10.1080/15230430.2022.2118639.

[42] Walter Anthony KM, Anthony P, Hasson N, Edgar C, Sivan O, Eliani-Russak E, Bergman O, Minsley BJ, James SR, Pastick NJ, Kholodov A, Zimov S, Euskirchen E, Bret-Harte MS, Grosse G, Langer M, Nitzbon J. Upland Yedoma taliks are an unpredicted source of atmospheric methane. Nat Commun. 2024;15:6056. 10.1038/s41467-024-50346-5. 39025864 10.1038/s41467-024-50346-5 PMC11258132

[43] Schädel C, Rogers BM, Lawrence DM, Koven CD, Brovkin V, Burke EJ, Genet H, Huntzinger DN, Jafarov E, McGuire AD, Riley WJ, Natali SM. Earth system models must include permafrost carbon processes. Nat Clim Change. 2024;14:114–6. 10.1038/s41558-023-01909-9.

[44] Meredith M, Sommerkorn M, Cassotta S, Derksen C, Ekaykin A, Hollowed A, Kofinas G, Mackintosh A, Melbourne-Thomas J, Muelbert MMC, Ottersen G, Pritchard H, Schuur EAG. Polar regions. In: Pörtner H-O, Roberts DC, Masson-Delmotte V, Zhai P, Tignor M, Poloczanska E, Mintenbeck K, Alegría A, Nicolai M, Okem A, Petzold J, Rama B, Weyer NM, editors. IPCC special report on the ocean and cryosphere in a changing climate. Cambridge, UK and New York: Cambridge University Press; 2019. p. 203–320. 10.1017/9781009157964.005.

[45] Schuur EAG, Bockheim J, Canadell JG, Euskirchen E, Field CB, Goryachkin SV, Hagemann S, Kuhry P, Lafleur PM, Lee H, Mazhitova G, Nelson FE, Rinke A, Romanovsky VE, Shiklomanov N, Tarnocai C, Venevsky S, Vogel JG, Zimov SA. Vulnerability of permafrost carbon to climate change: implications for the global carbon cycle. Bioscience. 2008;58:701–14. 10.1641/B580807.

[46] Schuur EAG, Abbott B. High risk of permafrost thaw. Nature. 2011;480:32–3. 10.1038/480032a. 22129707 10.1038/480032a

[47] Schneider von Deimling T, Grosse G, Strauss J, Schirrmeister L, Morgenstern A, Schaphoff S, Meinshausen M, Boike J. Observation-based modelling of permafrost carbon fluxes with accounting for deep carbon deposits and thermokarst activity. Biogeosciences. 2015;12:3469–88. 10.5194/bg-12-3469-2015.

[48] Turner MG, Calder WJ, Cumming GS, Hughes TP, Jentsch A, LaDeau SL, Lenton TM, Shuman BN, Turetsky MR, Ratajczak Z, Williams JW, Williams AP, Carpenter SR. Climate change, ecosystems and abrupt change: science priorities. Philos Trans R Soc B Biol Sci. 2020;375:20190105. 10.1098/rstb.2019.0105. 10.1098/rstb.2019.0105 PMC7017767 31983326

[49] Peters DPC, Lugo AE, Chapin FS, Pickett STA, Duniway M, Rocha AV, Swanson FJ, Laney C, Jones J. Cross-system comparisons elucidate disturbance complexities and generalities. Ecosphere. 2011;2:art81. 10.1890/ES11-00115.1.

[50] Sousa WP. The role of disturbance in natural communities. Annu Rev Ecol Syst. 1984;15:353–91. 10.1146/annurev.es.15.110184.002033.

[51] The ecology of natural disturbance and patch dynamics. Elsevier; 1985. 10.1016/C2009-0-02952-3. 10.1126/science.230.4724.434 17816073

[52] Turner MG. Disturbance and landscape dynamics in a changing world. Ecology. 2010;91:2833–49. 10.1890/10-0097.1. 21058545 10.1890/10-0097.1

[53] Jentsch A, White P. A theory of pulse dynamics and disturbance in ecology. Ecology. 2019;100: e02734. 10.1002/ecy.2734. 31018013 10.1002/ecy.2734 PMC6851700

[54] Smith MD, Knapp AK, Collins SL. A framework for assessing ecosystem dynamics in response to chronic resource alterations induced by global change. Ecology. 2009;90:3279–89. 10.1890/08-1815.1. 20120798 10.1890/08-1815.1

[55] Sayedi SS, Abbott BW, Vannière B, Leys B, Colombaroli D, Romera GG, Słowiński M, Aleman JC, Blarquez O, Feurdean A, Brown K, Aakala T, Alenius T, Allen K, Andric M, Bergeron Y, Biagioni S, Bradshaw R, Bremond L, Brisset E, Brooks J, Brugger SO, Brussel T, Cadd H, Cagliero E, Carcaillet C, Carter V, Catry FX, Champreux A, Chaste E, Chavardès RD, Chipman M, Conedera M, Connor S, Constantine M, Courtney Mustaphi C, Dabengwa AN, Daniels W, De Boer E, Dietze E, Estrany J, Fernandes P, Finsinger W, Flantua SGA, Fox-Hughes P, Gaboriau DM, Gayo ME, Girardin Martin P, Glenn J, Glückler R, González-Arango C, Groves M, Hamilton DS, Hamilton RJ, Hantson S, Hapsari KA, Hardiman M, Hawthorne D, Hoffman K, Inoue J, Karp AT, Krebs P, Kulkarni C, Kuosmanen N, Lacourse T, Ledru M-P, Lestienne M, Long C, López-Sáez JA, Loughlin N, Niklasson M, Madrigal J, Maezumi SY, Marcisz K, Mariani M, McWethy D, Meyer G, Molinari C, Montoya E, Mooney S, Morales-Molino C, Morris J, Moss P, Oliveras I, Pereira JM, Pezzatti GB, Pickarski N, Pini R, Rehn E, Remy CC, Revelles J, Rius D, Robin V, Ruan Y, Rudaya N, Russell-Smith J, Seppä H, Shumilovskikh L, Sommers TW, Tavşanoğlu Ç, Umbanhowar C, Urquiaga E, Urrego D, Vachula RS, Wallenius T, You C, Daniau A-L. Assessing changes in global fire regimes. Fire Ecol. 2024;20:18. 10.1186/s42408-023-00237-9.

[56] Clark PU, Pisias NG, Stocker TF, Weaver AJ. The role of the thermohaline circulation in abrupt climate change. Nature. 2002;415:863–9. 10.1038/415863a. 11859359 10.1038/415863a

[57] Overpeck JT, Cole JE. Abrupt change in earth’s climate system. Annu Rev Environ Resour. 2006;31:1–31. 10.1146/annurev.energy.30.050504.144308.

[58] Alley RB, Marotzke J, Nordhaus WD, Overpeck JT, Peteet DM, Pielke RA, Pierrehumbert RT, Rhines PB, Stocker TF, Talley LD, Wallace JM. Abrupt climate change. Science. 2003;299:2005–10. 10.1126/science.1081056. 12663908 10.1126/science.1081056

[59] Brovkin V, Brook E, Williams JW, Bathiany S, Lenton TM, Barton M, DeConto RM, Donges JF, Ganopolski A, McManus J, Praetorius S, De Vernal A, Abe-Ouchi A, Cheng H, Claussen M, Crucifix M, Gallopín G, Iglesias V, Kaufman DS, Kleinen T, Lambert F, Van Der Leeuw S, Liddy H, Loutre M-F, McGee D, Rehfeld K, Rhodes R, Seddon AWR, Trauth MH, Vanderveken L, Yu Z. Past abrupt changes, tipping points and cascading impacts in the Earth system. Nat Geosci. 2021;14:550–8. 10.1038/s41561-021-00790-5.

[60] Crowley TJ, North GR. Abrupt climate change and extinction events in earth history. Science. 1988;240:996–1002. 10.1126/science.240.4855.996. 17731712 10.1126/science.240.4855.996

[61] Botta F, Dahl-Jensen D, Rahbek C, Svensson A, Nogués-Bravo D. Abrupt change in climate and biotic systems. Curr Biol. 2019;29:R1045–54. 10.1016/j.cub.2019.08.066. 31593663 10.1016/j.cub.2019.08.066

[62] Pasher J, Seed E, Duffe J. Development of boreal ecosystem anthropogenic disturbance layers for Canada based on 2008 to 2010 Landsat imagery. Can J Remote Sens. 2013;39:42–58. 10.5589/m13-007.

[63] Dabros A, Pyper M, Castilla G. Seismic lines in the boreal and arctic ecosystems of North America: environmental impacts, challenges, and opportunities. Environ Rev. 2018;26:214–29. 10.1139/er-2017-0080.

[64] Devoie É, Connon RF, Beddoe R, Goordial J, Quinton WL, Craig JR. Disconnected active layers and unfrozen permafrost: a discussion of permafrost-related terms and definitions. Sci Total Environ. 2024;912:169017. 10.1016/j.scitotenv.2023.169017. 38040371 10.1016/j.scitotenv.2023.169017

[65] Gibson CM, Chasmer LE, Thompson DK, Quinton WL, Flannigan MD, Olefeldt D. Wildfire as a major driver of recent permafrost thaw in boreal peatlands. Nat Commun. 2018;9:3041. 10.1038/s41467-018-05457-1. 30072751 10.1038/s41467-018-05457-1 PMC6072743

[66] Braun KN, Andresen CG. Heterogeneity in ice-wedge permafrost degradation revealed across spatial scales. Remote Sens Environ. 2024;311:114299. 10.1016/j.rse.2024.114299.

[67] Coulombe S, Fortier D, Lacelle D, Kanevskiy M, Shur Y. Origin, burial and preservation of late Pleistocene-age glacier ice in Arctic permafrost (Bylot Island, NU, Canada). Cryosphere. 2019;13:97–111. 10.5194/tc-13-97-2019.

[68] Ohara N, Jones BM, Parsekian AD, Hinkel KM, Yamatani K, Kanevskiy M, Rangel RC, Breen AL, Bergstedt H. A new Stefan equation to characterize the evolution of thermokarst lake and talik geometry. Cryosphere. 2022;16:1247–64. 10.5194/tc-16-1247-2022.

[69] Meisel OH, Dean JF, Vonk JE, Wacker L, Reichart G-J, Dolman H. Porewater δ13CDOC indicates variable extent of degradation in different talik layers of coastal Alaskan thermokarst lakes. Biogeosciences. 2021;18:2241–58. 10.5194/bg-18-2241-2021.

[70] Chuvilin EM, Sokolova NS, Bukhanov BA, Davletshina DA, Spasennykh MY. Formation of gas-emission craters in Northern West Siberia: shallow controls. Geosciences. 2021;11:393. 10.3390/geosciences11090393.

[71] Schurmeier LR, Brouwer GE, Fagents SA. Formation of the Siberian Yamal gas emission crater via accumulation and explosive release of gas within permafrost. Permafr Periglac Process. 2024;35:33–45. 10.1002/ppp.2211.

[72] Morgado AMO, Rocha LAM, Cartwright JHE, Cardoso SSS. Osmosis drives explosions and methane release in Siberian permafrost. Geophys Res Lett. 2024;51:e2024GL108987. 10.1029/2024GL108987.

[73] Dinneen J. Siberia’s mysterious exploding craters may be caused by hot gas. In: New Sci. 2024. https://www.newscientist.com/article/2412072-siberias-mysterious-exploding-craters-may-be-caused-by-hot-gas/. Accessed 19 Mar 2024.

[74] Gray R. The mystery of Siberia’s exploding craters. In: BBC. 2020. https://www.bbc.com/future/article/20201130-climate-change-the-mystery-of-siberias-explosive-craters. Accessed 19 Mar 2024.

[75] Nemo L. Massive craters in Siberia are exploding into existence. What’s causing them? In: Discov. Mag. 2021. https://www.discovermagazine.com/environment/massive-craters-in-siberia-are-exploding-into-existence-whats-causing-them. Accessed 19 Mar 2024.

[76] Kizyakov A, Leibman M, Zimin M, Sonyushkin A, Dvornikov Y, Khomutov A, Dhont D, Cauquil E, Pushkarev V, Stanilovskaya Y. Gas emission craters and mound-predecessors in the North of West Siberia, similarities and differences. Remote Sens. 2020;12:2182. 10.3390/rs12142182.

[77] Walter Anthony K, Daanen R, Anthony P, Schneider Von Deimling T, Ping C-L, Chanton JP, Grosse G. Methane emissions proportional to permafrost carbon thawed in Arctic lakes since the 1950s. Nat Geosci. 2016;9:679–82. 10.1038/ngeo2795.

[78] Etiope G, Klusman RW. Geologic emissions of methane to the atmosphere. Chemosphere. 2002;49:777–89. 10.1016/S0045-6535(02)00380-6. 12430657 10.1016/s0045-6535(02)00380-6

[79] Tank SE, Vonk JE, Walvoord MA, McClelland JW, Laurion I, Abbott BW. Landscape matters: predicting the biogeochemical effects of permafrost thaw on aquatic networks with a state factor approach. Permafr Periglac Process. 2020. 10.1002/ppp.2057.

[80] Pelletier N, Talbot J, Olefeldt D, Turetsky M, Blodau C, Sonnentag O, Quinton WL. Influence of Holocene permafrost aggradation and thaw on the paleoecology and carbon storage of a peatland complex in northwestern Canada. The Holocene. 2017;27:1391–405. 10.1177/0959683617693899.

[81] Walter KM, Smith LC, Stuart Chapin F. Methane bubbling from northern lakes: present and future contributions to the global methane budget. Philos Trans R Soc Math Phys Eng Sci. 2007;365:1657–76. 10.1098/rsta.2007.2036. 10.1098/rsta.2007.2036 17513268

[82] Beamish A, Neil A, Wagner I, Scott NA. Short-term impacts of active layer detachments on carbon exchange in a high arctic ecosystem, Cape Bounty, Nunavut, Canada. Polar Biol. 2014;37:1459–68. 10.1007/s00300-014-1536-4.

[83] Pautler BG, Simpson AJ, Mcnally DJ, Lamoureux SF, Simpson MJ. Arctic permafrost active layer detachments stimulate microbial activity and degradation of soil organic matter. Environ Sci Technol. 2010;44:4076–82. 10.1021/es903685j. 20459054 10.1021/es903685j

[84] Parmentier FW, Nilsen L, Tømmervik H, Meisel OH, Bröder L, Vonk JE, Westermann S, Semenchuk PR, Cooper EJ. Rapid ice-wedge collapse and permafrost carbon loss triggered by increased snow depth and surface runoff. Geophys Res Lett. 2024;51:e2023GL108020. 10.1029/2023GL108020.

[85] Anthony KMW, Zimov SA, Grosse G, Jones MC, Anthony PM, Iii FSC, Finlay JC, Mack MC, Davydov S, Frenzel P, Frolking S. A shift of thermokarst lakes from carbon sources to sinks during the Holocene epoch. Nature. 2014;511:452–6. 10.1038/nature13560. 25043014 10.1038/nature13560

[86] Payette S, Delwaide A, Caccianiga M, Beauchemin M. Accelerated thawing of subarctic peatland permafrost over the last 50 years. Geophys Res Lett. 2004;31:2004GL020358. 10.1029/2004GL020358.

[87] Jones MC, Harden J, O’Donnell J, Manies K, Jorgenson T, Treat C, Ewing S. Rapid carbon loss and slow recovery following permafrost thaw in boreal peatlands. Glob Change Biol. 2017;23:1109–27. 10.1111/gcb.13403. 10.1111/gcb.13403 27362936

[88] Yoshikawa K, Bolton WR, Romanovsky VE, Fukuda M, Hinzman LD. Impacts of wildfire on the permafrost in the boreal forests of Interior Alaska. J Geophys Res Atmospheres. 2002;107. 10.1029/2001JD000438.

[89] Smith SL, Riseborough DW, Bonnaventure PP. Eighteen year record of forest fire effects on ground thermal regimes and permafrost in the central Mackenzie Valley, NWT, Canada. Permafr Periglac Process. 2015;26:289–303. 10.1002/ppp.1849.

[90] Baltzer JL, Veness T, Chasmer LE, Sniderhan AE, Quinton WL. Forests on thawing permafrost: fragmentation, edge effects, and net forest loss. Glob Change Biol. 2014;20:824–34. 10.1111/gcb.12349. 10.1111/gcb.12349 23939809

[91] Painter SL, Coon ET, Khattak AJ, Jastrow JD. Drying of tundra landscapes will limit subsidence-induced acceleration of permafrost thaw. Proc Natl Acad Sci. 2023;120:e2212171120. 10.1073/pnas.2212171120. 36780526 10.1073/pnas.2212171120 PMC9974406

[92] Barth S, Nitze I, Juhls B, Runge A, Grosse G. Rapid changes in retrogressive thaw slump dynamics in the Russian high arctic based on very high-resolution remote sensing. Geophys Res Lett. 2025;52:e2024GL113022. 10.1029/2024GL113022.

[93] Yang Y, Rogers BM, Fiske G, Watts J, Potter S, Windholz T, Mullen A, Nitze I, Natali SM. Mapping retrogressive thaw slumps using deep neural networks. Remote Sens Environ. 2023;288:113495. 10.1016/j.rse.2023.113495.

[94] Nitze I, Heidler K, Barth S, Grosse G. Developing and testing a deep learning approach for mapping retrogressive thaw slumps. Remote Sens. 2021;13:4294. 10.3390/rs13214294.

[95] Kokelj SV, Gingras-Hill T, Daly SV, Morse P, Wolfe S, Rudy ACA, Van Der Sluijs J, Weiss N, O’Neill B, Baltzer J, Lantz TC, Gibson C, Cazon D, Fraser RH, Froese DG, Giff G, Klengenberg C, Lamoureux SF, Quinton W, Turetsky MR, Chiasson A, Ferguson C, Newton M, Pope M, Paul JA, Wilson A, Young J. The Northwest territories thermokarst mapping collective: a northern-driven mapping collaborative toward understanding the effects of permafrost thaw. Arct Sci. 2023;9(4):886–918. 10.1139/AS-2023-0009.

[96] Lawrence DM, Koven CD, Swenson SC, Riley WJ, Slater AG. Permafrost thaw and resulting soil moisture changes regulate projected high-latitude CO2 and CH4 emissions. Environ Res Lett. 2015;10:094011. 10.1088/1748-9326/10/9/094011.

[97] Jones MC, Grosse G, Treat C, Turetsky M, Anthony KW, Brosius L. Past permafrost dynamics can inform future permafrost carbon-climate feedbacks. Commun Earth Environ. 2023;4:272. 10.1038/s43247-023-00886-3.

[98] Brosius LS, Anthony KMW, Treat CC, Lenz J, Jones MC, Bret-Harte MS, Grosse G. Spatiotemporal patterns of northern lake formation since the last glacial maximum. Quat Sci Rev. 2021;253:106773. 10.1016/j.quascirev.2020.106773.

[99] Fuchs M, Lenz J, Jock S, Nitze I, Jones BM, Strauss J, Günther F, Grosse G. Organic carbon and nitrogen stocks along a thermokarst lake sequence in arctic Alaska. J Geophys Res Biogeosciences. 2019;124:1230–47. 10.1029/2018JG004591. 10.1029/2018JG004591 PMC6618060 31341754

[100] Li W, Hsu C-Y, Wang S, Gu Z, Yang Y, Rogers BM, Liljedahl A. A multi-scale vision transformer-based multimodal GeoAI model for mapping Arctic permafrost thaw. IEEE J Sel Top Appl Earth Observ Remote Sens. 2025:18:12209–23. 10.1109/JSTARS.2025.3564310.

[101] Li W, Arundel S, Gao S, Goodchild M, Hu Y, Wang S, Zipf A. GeoAI for science and the science of GeoAI. J Spat Inf Sci. 2024;1–17. 10.5311/JOSIS.2024.29.349.

[102] Elder CD, Thompson DR, Thorpe AK, Chandanpurkar HA, Hanke PJ, Hasson N, James SR, Minsley BJ, Pastick NJ, Olefeldt D, Walter Anthony KM, Miller CE. Characterizing methane emission hotspots from thawing permafrost. Glob Biogeochem Cycles. 2021;35:e2020GB006922. 10.1029/2020GB006922.

[103] Knoblauch C, Beer C, Schuett A, Sauerland L, Liebner S, Steinhof A, Rethemeyer J, Grigoriev MN, Faguet A, Pfeiffer E. Carbon dioxide and methane release following abrupt thaw of pleistocene permafrost deposits in arctic Siberia. J Geophys Res Biogeosciences. 2021;126. 10.1029/2021JG006543.

