# Supplementary material for: A Review of Abrupt Permafrost Thaw: Definitions, Usage, and a Proposed Conceptual Framework
Source: Curr Clim Change Rep. 2025 Jul 24;11(1):7. doi: 10.1007/s40641-025-00204-3 (PMC12289745; doi:10.1007/s40641-025-00204-3)
Supplement: Supplementary file 1 — Supplementary file1 (DOCX 288 KB) [file 40641_2025_204_MOESM1_ESM.docx]

# **Supplementary Information: A review of abrupt permafrost thaw: Definitions, usage, and a proposed conceptual framework**

**Hailey Webb^1,2^, Matthias Fuchs^1^, Benjamin W. Abbott^3^, Thomas A. Douglas^4^, Clayton D. Elder^5,6^, Jessica Gilman Ernakovich^7,8^, Eugenie S. Euskirchen^9^, Mathias Göckede^10^, Guido Grosse^11,12^, Gustaf Hugelius^13^, Miriam C. Jones^14^, Charles Koven^15^, Heather Kropp^16^, Emma Lathrop^17^, WenWen Li^18^, Michael M. Loranty^19^, Susan M. Natali^20^, David Olefeldt^21^, Christina Schädel^20^, Edward A.G. Schuur^17^, Oliver Sonnentag^22^, Jens Strauss^11^, Anna-Maria Virkkala^20^, Merritt R. Turetsky^1,2^**

^1^ Renewable and Sustainable Energy Institute, University of Colorado Boulder, Boulder, CO USA

^2^ Department of Ecology and Evolutionary Biology, University of Colorado Boulder, Boulder, CO USA

^3^ Department of Plant & Wildlife Sciences, Brigham Young University, Provo, UT USA

^4^ U.S. Army Cold Regions Research and Engineering Laboratory, Fort Wainwright, AK 99703 USA

^5^ Jet Propulsion Laboratory, California Institute of Technology, Pasadena, CA USA

^6^ Earth Sciences Division, NASA Ames Research Center, Moffett Field, CA USA

^7^ Center for Soil Biogeochemistry and Microbial Ecology, University of New Hampshire, Durham NH USA

^8^ Department of Natural Resources and the Environment, University of New Hampshire, Durham NH USA

^9^ Institute of Arctic Biology, University of Alaska Fairbanks, Fairbanks, AK USA 99775

^10^ Max Planck Institute for Biogeochemistry, Jena, Germany

^11^ Alfred Wegener Institute Helmholtz Centre for Polar and Marine Research, Permafrost Research Section, 14473 Potsdam, Germany

^12^ University of Potsdam, Institute of Geoscience, 14476 Potsdam, Germany

^13^ Department of Physical Geography and Bolin Centre for Climate Research, Stockholm University

^14^ Florence Bascom Geoscience Center, U.S. Geological Survey, Reston, VA 20192

^15^ Earth Climate & Ecosystems Sciences, Lawrence Berkeley National Lab, Berkeley, CA USA 94720

^16^ Environmental Studies Program, Hamilton College, Clinton, NY USA

^17^ Center for Ecosystem Science and Society, Northern Arizona University, Flagstaff, AZ 86001, USA

^18^ School of Geographical Sciences and Urban Planning, Arizona State University, Tempe, AZ, USA 85287-5302

^19^ Department of Geography, Colgate University, Hamilton, NY USA

^20^ Woodwell Climate Research Center, Falmouth, MA 02540 USA

^21^ Department of Renewable Resources, University of Alberta, Edmonton, Canada

^22^ Université de Montréal, Département de géograhie, Montréal, QC H3C 3J7, Canada

**Corresponding Author:**

**Hailey Webb**

hailey.webb@colorado.edu

| **Source** | **Definition of abrupt thaw** |
| --- | --- |
| Jorgenson et al. 2006 [1] | Rate of thaw |
| Schuur et al. 2008 [2] | Rate of thaw |
| Osterkamp and Jorgenson 2009 [3] | Rate of thaw |
| Schuur and Abbott 2011 [4] | Thermokarst/thermal erosion |
| Grosse et al. 2011 [5] | Mixed |
| Schuur et al. 2013 [6] | Thermokarst/thermal erosion |
| Schädel et al. 2014 [7] | Thermokarst/thermal erosion |
| Schneider von Deimling et al. 2015 [8] | Rate of thaw |
| Schuur et al. 2015 [9] | Thermokarst/thermal erosion |
| Ping et al. 2015 [10] | Rate of thaw |
| Ewing et al. 2015 [11] | Thermokarst/thermal erosion |
| Abbott et al. 2015 [12] | Thermokarst/thermal erosion |
| Bouchard et al. 2015 [13] | Thermokarst/thermal erosion |
| Lique et al. 2015 [14] | Rate of thaw |
| van der Kolk 2016 [15] | Thermokarst/thermal erosion |
| Schädel et al. 2016 [16] | Mixed |
| Zhu et al. 2016 [17] | Rate of thaw |
| Trubl et al. 2016 [18] | Rate of thaw |
| Martin 2016 [19] | Thermokarst/thermal erosion |
| Loiko et al. 2017 [20] | Mixed |
| Voigt et al. 2017 [21] | Thermokarst/thermal erosion |
| Mamet et al. 2017 [22] | Thermokarst/thermal erosion |
| Loisel et al. 2017 [23] | Rate of thaw |
| Li et al. 2017 [24] | Mixed |
| Strauss et al. 2017 [25] | Mixed |
| Vincent et al. 2017 [26] | Rate of thaw |
| Lin & Biswas 2017 [27] | Mixed |
| Water Anthony et al. 2018 [28] | Mixed |
| Schuur & Mack 2018 [29] | Mixed |
| Malhotra et al. 2018 [30] | Rate of thaw |
| Parazoo et al. 2018 [31] | Thermokarst/thermal erosion |
| Liu et al. 2018 [32] | Thermokarst/thermal erosion |
| Chen et al. 2018 [33] | Rate of thaw |
| Schneider von Deimling et al. 2018 [34] | Mixed |
| Schuur et al. 2018 [35] | Mixed |
| Ramage et al. 2018 [36] | Thermokarst/thermal erosion |
| Dean et al. 2018 [37] | Thermokarst/thermal erosion |
| Heslop et al. 2019 [38] | Thermokarst/thermal erosion |
| Wilkerson et al. 2019 [39] | Thermokarst/thermal erosion |
| Kurylyk 2019 [40] | Rate of thaw |
| Vonk et al. 2019 [41] | Mixed |
| Spring et al. 2019 [42] | Mixed |
| Runge & Grosse 2019 [43] | Rate of thaw |
| Vigneron et al. 2019 [44] | Mixed |
| Meredith et al. 2019 [45] | Mixed |
| Schaefer 2019 [46] | Mixed |
| Turetsky et al. 2020 [47] | Mixed |
| Opfergelt et al. 2020 [48] | Mixed |
| Douglas et al. 2020 [49] | Mixed |
| Van Huissteden 2020 [50] | Thermokarst/thermal erosion |
| Li et al. 2020 [51] | Thermokarst/thermal erosion |
| Rodenhizer et al. 2020 [52] | Mixed |
| Wang et al. 2020 [53] | Mixed |
| Magnússon et al. 2020 [54] | Thermokarst/thermal erosion |
| Nitzbon et al. 2020 [55] | Rate of thaw |
| Voigt et al. 2020 [56] | Mixed |
| Van Huissteden 2020 [57] | Mixed |
| Mu et al. 2020 [58] | Thermokarst/thermal erosion |
| Lapham et al. 2020 [59] | Rate of thaw |
| Jong et al. 2020 [60] | Rate of thaw |
| Burke et al. 2020 [61] | Thermokarst/thermal erosion |
| Bröder et al. 2020 [62] | Thermokarst/thermal erosion |
| Runge & Grosse 2020 [63] | Thermokarst/thermal erosion |
| Dean et al. 2020 [64] | Thermokarst/thermal erosion |
| Jones et al. 2020 [65] | Mixed |
| Wang et al. 2020 [66] | Mixed |
| in ‘t Zandt et al. 2020 [67] | Rate of thaw |
| Macias-Fauria et al. 2020 [68] | Mixed |
| Ahmad et al. 2020 [69] | Mixed |
| Bhuiyan et al. 2020 [70] | Thermokarst/thermal erosion |
| Bengston et al. 2020 [71] | Rate of thaw |
| Marques et al. 2020 [72] | Rate of thaw |
| Dabrowski et al. 2020 [73] | Rate of thaw |
| Goudie 2020 [74] | Thermokarst/thermal erosion |
| Meinshausen et al. 2020 [75] | Thermokarst/thermal erosion |
| Hugelius et al. 2020 [76] | Mixed |
| Tank et al. 2020 [77] | Rate of thaw |
| Gagné et al. 2020 [78] | Thermokarst/thermal erosion |
| Knoblauch et al. 2021 [79] | Thermokarst/thermal erosion |
| Natali et al. 2021 [80] | Mixed |
| Walter Anthony et al. 2021 [81] | Mixed |
| Gibson et al. 2021 [82] | Mixed |
| Anisimov & Zimov 2021 [83] | Rate of thaw |
| Elder et al. 2021 [84] | Thermokarst/thermal erosion |
| Jin et al. 2021 [85] | Mixed |
| Treat et al. 2021 [86] | Mixed |
| Miner et al. 2021 [87] | Rate of thaw |
| Waldrop et al. 2021 [88] | Mixed |
| Kuhn et al. 2021 [89] | Mixed |
| Marushchak et al. 2021 [90] | Rate of thaw |
| Bröder et al. 2021 [91] | Mixed |
| Strauss et al. 2021 [92] | Thermokarst/thermal erosion |
| Keskitalo et al. 2021 [93] | Mixed |
| MacDougall 2021 [94] | Mixed |
| Gibson et al. 2021 [95] | Mixed |
| Siewert et al. 2021 [96] | Thermokarst/thermal erosion |
| Chen et al. 2021 [97] | Mixed |
| Woodard et al. 2021 [98] | Mixed |
| Rogers et al. 2021 [99] | Thermokarst/thermal erosion |
| Jones et al. 2021 [100] | Rate of thaw |
| Wologo et al. 2021 [101] | Thermokarst/thermal erosion |
| Abbott et al. 2021 [102] | Mixed |
| Magnússon et al. 2021 [103] | Thermokarst/thermal erosion |
| Christensen et al. 2021 [104] | Thermokarst/thermal erosion |
| Pfeiffer et al. 2021 [105] | Thermokarst/thermal erosion |
| Behnke et al. 2021 [106] | Thermokarst/thermal erosion |
| Jongejans et al. 2021 [107] | Thermokarst/thermal erosion |
| Monhonval et al. 2021 [108] | Mixed |
| Yin et al. 2021 [109] | Rate of thaw |
| Bruhwiler et al. 2021 [110] | Mixed |
| Philipp et al. 2021 [111] | Mixed |
| Andresen & Lougheed et al. 2021 [112] | Mixed |
| Liu et al. 2021 [113] | Mixed |
| Slaymaker et al. 2021 [114] | Mixed |
| Van Huissteden et al. 2021 [115] | Thermokarst/thermal erosion |
| Jandt et al. 2021 [116] | Rate of thaw |
| Scheller et al. 2021 [117] | Thermokarst/thermal erosion |
| Miner et al. 2022 [118] | Rate of thaw |
| Rodenhizer et al. 2022 [119] | Thermokarst/thermal erosion |
| Heijmans et al. 2022 [120] | Rate of thaw |
| Runge et al. 2022 [121] | Mixed |
| Webb et al. 2022 [122] | Mixed |
| Ernakovich et al. 2022 [123] | Mixed |
| Pellerin et al. 2022 [124] | Mixed |
| Minsley et al. 2022 [125] | Rate of thaw |
| Hjort et al. 2022 [126] | Thermokarst/thermal erosion |
| Scheel et al. 2022 [127] | Mixed |
| Wegner et al. 2022 [128] | Thermokarst/thermal erosion |
| Burke et al. 2022 [129] | Thermokarst/thermal erosion |
| Strauss et al. 2022 [130] | Mixed |
| Schuur et al. 2022 [131] | Thermokarst/thermal erosion |
| Chen et al. 2022 [132] | Mixed |
| Treharne et al. 2022 [133] | Mixed |
| Abbott 2022 [134] | Thermokarst/thermal erosion |
| Hirst et al. 2022 [135] | Mixed |
| Chen et al. 2022 [136] | Rate of thaw |
| Wang et al. 2022 [137] | Mixed |
| Patzner et al. 2022 [138] | Mixed |
| Bernhard et al. 2022 [139] | Mixed |
| Treat et al. 2022 [140] | Mixed |
| Wei et al. 2022 [141] | Thermokarst/thermal erosion |
| Witharana et al. 2022 [142] | Mixed |
| Smith et al. 2022 [143] | Thermokarst/thermal erosion |
| Natali et al. 2022 [144] | Thermokarst/thermal erosion |
| Fiencke et al. 2022 [145] | Rate of thaw |
| Korosi et al. 2022 [146] | Thermokarst/thermal erosion |
| Freitas et al. 2022 [147] | Thermokarst/thermal erosion |
| Jongejans et al. 2022 [148] | Thermokarst/thermal erosion |
| Shakil et al. 2022 [149] | Rate of thaw |
| Tape et al. 2022 [150] | Mixed |
| Del Vecchio et al. 2022 [151] | Rate of thaw |
| Keskitalo et al. 2022 [152] | Mixed |
| Baskaran et al. 2022 [153] | Mixed |
| Armstrong Mckay et al. 2022 [154] | Thermokarst/thermal erosion |
| Kuhry et al. 2022 [155] | Thermokarst/thermal erosion |
| van Oort et al. 2022 [156] | Mixed |
| Cuesta-Valero et al. 2023 [157] | Rate of thaw |
| Rodenhizer et al. 2023 [158] | Mixed |
| Painter et al. 2023 [159] | Rate of thaw |
| Scheel et al. 2023 [160] | Mixed |
| Bartsch et al. 2023 [161] | Mixed |
| Jones et al. 2023 [162] | Mixed |
| Mu et al. 2023 [163] | Rate of thaw |
| Monhonval et al. 2023 [164] | Thermokarst/thermal erosion |
| Miner et al. 2023 [165] | Thermokarst/thermal erosion |
| Gay et al. 2023 [166] | Thermokarst/thermal erosion |
| Beer et al. 2023 [167] | Mixed |
| Thomas et al. 2023 [168] | Thermokarst/thermal erosion |
| Murton et al. 2023 [169] | Rate of thaw |
| Kemeny et al. 2023 [170] | Thermokarst/thermal erosion |
| Yang et al. 2023 [171] | Mixed |
| Jiao et al. 2023 [172] | Mixed |
| Verfaillie et al. 2023 [173] | Rate of thaw |
| Brosius et al. 2023 [174] | Mixed |
| Arndt et al. 2023 [175] | Thermokarst/thermal erosion |
| Wang et al. 2023 [176] | Mixed |
| Wagner et al. 2023 [177] | Mixed |
| Sjöberg et al. 2023 [178] | Thermokarst/thermal erosion |
| Winkelmann et al. 2023 [179] | Mixed |
| Palazzo Corner et al. 2023 [180] | Thermokarst/thermal erosion |
| Shannon et al. 2023 [181] | Thermokarst/thermal erosion |
| Saros et al. 2023 [182] | Mixed |
| Hessen et al. 2024 [183] | Mixed |
| Nitzbon et al. 2024 [184] | Mixed |
| Parmentier et al. 2024 [185] | Mixed |
| Ramage et al. 2024 [186] | Mixed |
| Zhu et al. 2024 [187] | Rate of thaw |
| Treat et al. 2024 [188] | Mixed |
| Yang et al. 2024 [189] | Mixed |
| Schädel et al. 2024 [190] | Thermokarst/thermal erosion |
| García-Palacios 2024 [191] | Rate of thaw |
| Valman et al. 2024 [192] | Thermokarst/thermal erosion |
| Gao et al. 2024 [193] | Thermokarst/thermal erosion |
| Jiao et al. 2024 [194] | Mixed |
| Makopoulou et al. 2024 [195] | Thermokarst/thermal erosion |
| Miner et al. 2024 [196] | Mixed |
| Mu et al. 2024 [197] | Thermokarst/thermal erosion |
| Sabino et al. 2024 [198] | Rate of thaw |
| Strauss et al. 2024 [199] | Mixed |
| Strauss et al. 2024 [200] | Rate of thaw |
| Hugelius et al. 2024 [201] | Mixed |
| Rodenhizer et al. 2024 [202] | Mixed |
| Yang et al. 2024 [203] | Thermokarst/thermal erosion |
| Thomas et al. 2024 [204] | Mixed |
| Carneiro Barreto et al. 2024 [205] | Rate of thaw |
| Liljedahl et al. 2024 [206] | Mixed |
| Fouché et al. 2024 [207] | Thermokarst/thermal erosion |
| Ackermann et al. 2024 [208] | Mixed |
| Khattak et al. 2024 [209] | Mixed |
| Walter Anthony et al. 2024 [210] | Thermokarst/thermal erosion |
| Lenton et al. 2024 [211] | Rate of thaw |
| Smith et al. 2024 [212] | Rate of thaw |
| Huang et al. 2024 [213] | Mixed |
| Zhou et al. 2024 [214] | Mixed |
| Parmentier et al. 2024 [215] | Thermokarst/thermal erosion |
| Abernethy & Jackson et al. 2024 [216] | Mixed |
| Jenrich et al. 2024 [217] | Rate of thaw |
| Keskitalo et al. 2024 [218] | Mixed |
| Liu et al. 2024 [219] | Thermokarst/thermal erosion |
| Bartsch et al. 2024 [220] | Mixed |
| Anderson et al. 2024 [221] | Mixed |
| Mu et al. 2024 [222] | Mixed |
| Crumley et al. 2024 [223] | Thermokarst/thermal erosion |
| Gorham et al. 2024 [224] | Rate of thaw |
| Seiler et al. 2024 [225] | Mixed |
| Schimel et al. 2024 [226] | Mixed |

**Supplementary table 1.** List of all published journal articles, reports, and books that either explicitly or implicitly use the term or concept of abrupt thaw. The concept of abrupt thaw is usually generalized into one of three categories: 1) a rate of thaw; 2) used synonymously with thermokarst or thermal erosion; or 3) a mix of both a rate of thaw and thermokarst/thermal erosion.

| **Degree of ecosystem change classification** | **Description** |
| --- | --- |
| Low Severity | Linear change to the ecosystem that is temporary or does not have substantial impact on ecosystem identity or function |
| Moderate Severity | Linear change to the ecosystem that is temporary but has a substantial impact on ecosystem function |
| High Severity | Linear change to the ecosystem that is lasting with consequences for ecosystem identity and function |
| Complete Ecosystem State Change | Nonlinear change to the ecosystem that leads to long-term replacement of ecosystem identity and function |

**Supplementary table 2**. Definitions for each degree of ecosystem change classification. These categories were adapted from Turner et al. 2020 [227].

| **Permafrost Thaw Process** | **Rate of Change (years)** | | **Degree of Ecosystem Change (Table 1)** | **References** |
| --- | --- | --- | --- | --- |
|  | **Typical Change** | **Range** |  |  |
| Active layer detachment sliding | 0-9 | 0-99 | Complete State Change | [228–230] |
| Coastal erosion | 0-9 | 0-99 | Complete State Change | [231, 232] |
| Collapsing pingo | 10-99 | 0-99 | Moderate | [233, 234] |
| Gas-emission crater formation | 0-9 | 0-9 | Complete State Change | [235, 236] |
| Gelifluction | 100-999 | 10-999 | High | [237] |
| Gradual top-down thaw | 10-99 | 10-999 | Low | [238] |
| Retrogressive thaw slumping | 0-9 | 0-99 | Complete State Change | [239] |
| Riverbank erosion | 0-9 | 0-99 | Complete State Change | [240–242] |
| Talik formation | 10-99 | 0-99 | Moderate | [243–246] |
| Thermal erosion | 0-9 | 0-9 | High | [247–250] |
| Thermokarst lake formation | 10-99 | 10-999 | Complete State Change | [1, 251] |
| Thermokarst pits & trough formation | 10-99 | 0-99 | Moderate | [248, 252, 253] |
| Thermokarst water track formation | 0-9 | 0-99 | High | [254, 255] |
| Thermokarst wetland formation | 10-99 | 0-999 | High | [256–263] |
| Wildfire-induced top-down thaw | 0-9 | 0-99 | High | [116, 256, 264–270] |

**Supplementary table 3**. Summary of formation time and degree of ecosystem change for a subset of common permafrost thaw process.

**References**

1. Jorgenson MT, Shur YL, Pullman ER (2006) Abrupt increase in permafrost degradation in Arctic Alaska. Geophys Res Lett 33:L02503. https://doi.org/10.1029/2005GL024960

2. Schuur EAG, Bockheim J, Canadell JG, Euskirchen E, Field CB, Goryachkin SV, Hagemann S, Kuhry P, Lafleur PM, Lee H, Mazhitova G, Nelson FE, Rinke A, Romanovsky VE, Shiklomanov N, Tarnocai C, Venevsky S, Vogel JG, Zimov SA (2008) Vulnerability of Permafrost Carbon to Climate Change: Implications for the Global Carbon Cycle. BioScience 58:701–714. https://doi.org/10.1641/B580807

3. Osterkamp TE, Jorgenson MT (2009) Permafrost conditions and processes. In: Geological Monitoring. Geological Society of America

4. Schuur EAG, Abbott B (2011) High risk of permafrost thaw. Nature 480:32–33. https://doi.org/10.1038/480032a

5. Grosse G, Harden J, Turetsky M, McGuire AD, Camill P, Tarnocai C, Frolking S, Schuur EAG, Jorgenson T, Marchenko S, Romanovsky V, Wickland KP, French N, Waldrop M, Bourgeau-Chavez L, Striegl RG (2011) Vulnerability of high-latitude soil organic carbon in North America to disturbance. J Geophys Res 116:G00K06. https://doi.org/10.1029/2010JG001507

6. Schuur EAG, Abbott BW, Bowden WB, Brovkin V, Camill P, Canadell JG, Chanton JP, Chapin FS, Christensen TR, Ciais P, Crosby BT, Czimczik CI, Grosse G, Harden J, Hayes DJ, Hugelius G, Jastrow JD, Jones JB, Kleinen T, Koven CD, Krinner G, Kuhry P, Lawrence DM, McGuire AD, Natali SM, O’Donnell JA, Ping CL, Riley WJ, Rinke A, Romanovsky VE, Sannel ABK, Schädel C, Schaefer K, Sky J, Subin ZM, Tarnocai C, Turetsky MR, Waldrop MP, Walter Anthony KM, Wickland KP, Wilson CJ, Zimov SA (2013) Expert assessment of vulnerability of permafrost carbon to climate change. Clim Change 119:359–374. https://doi.org/10.1007/s10584-013-0730-7

7. Schädel C, Schuur EAG, Bracho R, Elberling B, Knoblauch C, Lee H, Luo Y, Shaver GR, Turetsky MR (2014) Circumpolar assessment of permafrost C quality and its vulnerability over time using long‐term incubation data. Glob Change Biol 20:641–652. https://doi.org/10.1111/gcb.12417

8. Schneider von Deimling T, Grosse G, Strauss J, Schirrmeister L, Morgenstern A, Schaphoff S, Meinshausen M, Boike J (2015) Observation-based modelling of permafrost carbon fluxes with accounting for deep carbon deposits and thermokarst activity. Biogeosciences 12:3469–3488. https://doi.org/10.5194/bg-12-3469-2015

9. Schuur EAG, McGuire AD, Schädel C, Grosse G, Harden JW, Hayes DJ, Hugelius G, Koven CD, Kuhry P, Lawrence DM, Natali SM, Olefeldt D, Romanovsky VE, Schaefer K, Turetsky MR, Treat CC, Vonk JE (2015) Climate change and the permafrost carbon feedback. Nature 520:171–179. https://doi.org/10.1038/nature14338

10. Ping CL, Jastrow JD, Jorgenson MT, Michaelson GJ, Shur YL (2015) Permafrost soils and carbon cycling. SOIL 1:147–171. https://doi.org/10.5194/soil-1-147-2015

11. Ewing SA, O’Donnell JA, Aiken GR, Butler K, Butman D, Windham‐Myers L, Kanevskiy MZ (2015) Long‐term anoxia and release of ancient, labile carbon upon thaw of Pleistocene permafrost. Geophys Res Lett 42:. https://doi.org/10.1002/2015GL066296

12. Abbott BW, Jones JB, Godsey SE, Larouche JR, Bowden WB (2015) Patterns and persistence of hydrologic carbon and nutrient export from collapsing upland permafrost. Biogeosciences 12:3725–3740. https://doi.org/10.5194/bg-12-3725-2015

13. Bouchard F, Laurion I, Pr&amp;#x0117;skienis V, Fortier D, Xu X, Whiticar MJ (2015) Modern to millennium-old greenhouse gases emitted from ponds and lakes of the Eastern Canadian Arctic (Bylot Island, Nunavut). Biogeosciences 12:7279–7298. https://doi.org/10.5194/bg-12-7279-2015

14. Lique C, Holland MM, Dibike YB, Lawrence DM, Screen JA (2016) Modeling the Arctic freshwater system and its integration in the global system: Lessons learned and future challenges. J Geophys Res Biogeosciences 121:540–566. https://doi.org/10.1002/2015JG003120

15. van der Kolk H-J, Heijmans MMPD, van Huissteden J, Pullens JWM, Berendse F (2016) Potential Arctic tundra vegetation shifts in response to changing temperature, precipitation and permafrost thaw. Biogeosciences 13:6229–6245. https://doi.org/10.5194/bg-13-6229-2016

16. Schädel C, Bader MK-F, Schuur EAG, Biasi C, Bracho R, Čapek P, De Baets S, Diáková K, Ernakovich J, Estop-Aragones C, Graham DE, Hartley IP, Iversen CM, Kane E, Knoblauch C, Lupascu M, Martikainen PJ, Natali SM, Norby RJ, O’Donnell JA, Chowdhury TR, Šantrůčková H, Shaver G, Sloan VL, Treat CC, Turetsky MR, Waldrop MP, Wickland KP (2016) Potential carbon emissions dominated by carbon dioxide from thawed permafrost soils. Nat Clim Change 6:950–953. https://doi.org/10.1038/nclimate3054

17. Zhu D, Peng S, Ciais P, Zech R, Krinner G, Zimov S, Grosse G (2016) Simulating soil organic carbon in yedoma deposits during the Last Glacial Maximum in a land surface model. Geophys Res Lett 43:5133–5142. https://doi.org/10.1002/2016GL068874

18. Trubl G, Solonenko N, Chittick L, Solonenko SA, Rich VI, Sullivan MB (2016) Optimization of viral resuspension methods for carbon-rich soils along a permafrost thaw gradient. PeerJ 4:e1999. https://doi.org/10.7717/peerj.1999

19. Martin C (2016) From death comes life. Curr Biol 26:R548–R552

20. Loiko SV, Pokrovsky OS, Raudina TV, Lim A, Kolesnichenko LG, Shirokova LS, Vorobyev SN, Kirpotin SN (2017) Abrupt permafrost collapse enhances organic carbon, CO2 , nutrient and metal release into surface waters. Chem Geol 471:153–165. https://doi.org/10.1016/j.chemgeo.2017.10.002

21. Voigt C, Marushchak ME, Lamprecht RE, Jackowicz-Korczyński M, Lindgren A, Mastepanov M, Granlund L, Christensen TR, Tahvanainen T, Martikainen PJ, Biasi C (2017) Increased nitrous oxide emissions from Arctic peatlands after permafrost thaw. Proc Natl Acad Sci 114:6238–6243. https://doi.org/10.1073/pnas.1702902114

22. Mamet SD, Chun KP, Kershaw GGL, Loranty MM, Peter Kershaw G (2017) Recent Increases in Permafrost Thaw Rates and Areal Loss of Palsas in the Western Northwest Territories, Canada. Permafr Periglac Process 28:619–633. https://doi.org/10.1002/ppp.1951

23. Loisel J, Van Bellen S, Pelletier L, Talbot J, Hugelius G, Karran D, Yu Z, Nichols J, Holmquist J (2017) Insights and issues with estimating northern peatland carbon stocks and fluxes since the Last Glacial Maximum. Earth-Sci Rev 165:59–80. https://doi.org/10.1016/j.earscirev.2016.12.001

24. Li B, Heijmans MMPD, Blok D, Wang P, Karsanaev SV, Maximov TC, Van Huissteden J, Berendse F (2017) Thaw pond development and initial vegetation succession in experimental plots at a Siberian lowland tundra site. Plant Soil 420:147–162. https://doi.org/10.1007/s11104-017-3369-8

25. Strauss J, Schirrmeister L, Grosse G, Fortier D, Hugelius G, Knoblauch C, Romanovsky V, Schädel C, Schneider von Deimling T, Schuur EAG, Shmelev D, Ulrich M, Veremeeva A (2017) Deep Yedoma permafrost: A synthesis of depositional characteristics and carbon vulnerability. Earth-Sci Rev 172:75–86. https://doi.org/10.1016/j.earscirev.2017.07.007

26. Vincent WF, Lemay M, Allard M (2017) Arctic permafrost landscapes in transition: towards an integrated Earth system approach. Arct Sci 3:39–64. https://doi.org/10.1139/as-2016-0027

27. Lin M, Biswas A (2017) Climate Mediated Changes in Permafrost and Their Effects on Natural and Human Environments. In: Rakshit A, Abhilash PC, Singh HB, Ghosh S (eds) Adaptive Soil Management : From Theory to Practices. Springer Singapore, Singapore, pp 477–512

28. Walter Anthony K, Schneider von Deimling T, Nitze I, Frolking S, Emond A, Daanen R, Anthony P, Lindgren P, Jones B, Grosse G (2018) 21st-century modeled permafrost carbon emissions accelerated by abrupt thaw beneath lakes. Nat Commun 9:3262. https://doi.org/10.1038/s41467-018-05738-9

29. Schuur EAG, Mack MC (2018) Ecological Response to Permafrost Thaw and Consequences for Local and Global Ecosystem Services. Annu Rev Ecol Evol Syst 49:279–301. https://doi.org/10.1146/annurev-ecolsys-121415-032349

30. Malhotra A, Moore TR, Limpens J, Roulet NT (2018) Post-thaw variability in litter decomposition best explained by microtopography at an ice-rich permafrost peatland. Arct Antarct Alp Res 50:e1415622. https://doi.org/10.1080/15230430.2017.1415622

31. Parazoo NC, Koven CD, Lawrence DM, Romanovsky V, Miller CE (2018) Detecting the permafrost carbon feedback: talik formation and increased cold-season respiration as precursors to sink-to-source transitions. The Cryosphere 12:123–144. https://doi.org/10.5194/tc-12-123-2018

32. Liu F, Chen L, Abbott BW, Xu Y, Yang G, Kou D, Qin S, Strauss J, Wang Y, Zhang B, Yang Y (2018) Reduced quantity and quality of SOM along a thaw sequence on the Tibetan Plateau. Environ Res Lett 13:104017. https://doi.org/10.1088/1748-9326/aae43b

33. Chen J, Liu L, Zhang T, Cao B, Lin H (2018) Using Persistent Scatterer Interferometry to Map and Quantify Permafrost Thaw Subsidence: A Case Study of Eboling Mountain on the Qinghai-Tibet Plateau. J Geophys Res Earth Surf 123:2663–2676. https://doi.org/10.1029/2018JF004618

34. Schneider Von Deimling T, Kleinen T, Hugelius G, Knoblauch C, Beer C, Brovkin V (2018) Long-term deglacial permafrost carbon dynamics in MPI-ESM. Clim Past 14:2011–2036. https://doi.org/10.5194/cp-14-2011-2018

35. Committee to Review the Draft Second State of the Carbon Cycle Report, Board on Atmospheric Sciences and Climate, Division on Earth and Life Studies, National Academies of Sciences, Engineering, and Medicine (2018) Review of the Draft Second State of the Carbon Cycle Report (SOCCR2). National Academies Press, Washington, D.C.

36. Ramage JL, Irrgang AM, Morgenstern A, Lantuit H (2018) Increasing coastal slump activity impacts the release of sediment and organic carbon into the Arctic Ocean. Biogeosciences 15:1483–1495. https://doi.org/10.5194/bg-15-1483-2018

37. Dean JF, Middelburg JJ, Röckmann T, Aerts R, Blauw LG, Egger M, Jetten MSM, De Jong AEE, Meisel OH, Rasigraf O, Slomp CP, In’T Zandt MH, Dolman AJ (2018) Methane Feedbacks to the Global Climate System in a Warmer World. Rev Geophys 56:207–250. https://doi.org/10.1002/2017RG000559

38. Heslop JK, Winkel M, Walter Anthony KM, Spencer RGM, Podgorski DC, Zito P, Kholodov A, Zhang M, Liebner S (2019) Increasing Organic Carbon Biolability With Depth in Yedoma Permafrost: Ramifications for Future Climate Change. J Geophys Res Biogeosciences 124:2021–2038. https://doi.org/10.1029/2018JG004712

39. Wilkerson J, Dobosy R, Sayres DS, Healy C, Dumas E, Baker B, Anderson JG (2019) Permafrost nitrous oxide emissions observed on a landscape scale using the airborne eddy-covariance method. Atmospheric Chem Phys 19:4257–4268. https://doi.org/10.5194/acp-19-4257-2019

40. Kurylyk BL (2019) Engineering challenges of warming. Nat Clim Change 9:807–808. https://doi.org/10.1038/s41558-019-0612-8

41. Vonk JE, Tank SE, Walvoord MA (2019) Integrating hydrology and biogeochemistry across frozen landscapes. Nat Commun 10:5377. https://doi.org/10.1038/s41467-019-13361-5

42. Spring A, Skinner K, Simba M, Nelson E, Baltzer J, Swanson H, Turetsky M (2019) Taking care of the land: An interdisciplinary approach to community-based food systems assessment in Kakisa, Northwest Territories, Canada. In: Sustainable Food System Assessment. Routledge, pp 42–65

43. Runge A, Grosse G (2019) Comparing Spectral Characteristics of Landsat-8 and Sentinel-2 Same-Day Data for Arctic-Boreal Regions. Remote Sens 11:1730. https://doi.org/10.3390/rs11141730

44. Vigneron A, Cruaud P, Bhiry N, Lovejoy C, Vincent WF (2019) Microbial Community Structure and Methane Cycling Potential along a Thermokarst Pond-Peatland Continuum. Microorganisms 7:486. https://doi.org/10.3390/microorganisms7110486

45. Meredith M, Cassotta S, Derksen C, Ekaykin A, Hollowed A, Kofinas G, Mackintosh A, Melbourne-Thomas J, M.C. Muelbert M, Ottersen G, Pritchard H, Schuur EAG (2019) Polar Regions In: IPCC Special Report on the Ocean and Cryosphere in a Changing Climate

46. Schaefer H (2019) On the Causes and Consequences of Recent Trends in Atmospheric Methane. Curr Clim Change Rep 5:259–274. https://doi.org/10.1007/s40641-019-00140-z

47. Turetsky MR, Abbott BW, Jones MC, Anthony KW, Olefeldt D, Schuur EAG, Grosse G, Kuhry P, Hugelius G, Koven C, Lawrence DM, Gibson C, Sannel ABK, McGuire AD (2020) Carbon release through abrupt permafrost thaw. Nat Geosci 13:138–143. https://doi.org/10.1038/s41561-019-0526-0

48. Opfergelt S (2020) The next generation of climate model should account for the evolution of mineral-organic interactions with permafrost thaw. Environ Res Lett 15:091003. https://doi.org/10.1088/1748-9326/ab9a6d

49. Douglas TA, Turetsky MR, Koven CD (2020) Increased rainfall stimulates permafrost thaw across a variety of Interior Alaskan boreal ecosystems. Npj Clim Atmospheric Sci 3:28. https://doi.org/10.1038/s41612-020-0130-4

50. Van Huissteden J (2020) Models: Forecasting the Present and Future of Permafrost. In: Thawing Permafrost. Springer International Publishing, Cham, pp 465–497

51. Li H, Väliranta M, Mäki M, Kohl L, Sannel ABK, Pumpanen J, Koskinen M, Bäck J, Bianchi F (2020) Overlooked organic vapor emissions from thawing Arctic permafrost. Environ Res Lett 15:104097. https://doi.org/10.1088/1748-9326/abb62d

52. Rodenhizer H, Ledman J, Mauritz M, Natali SM, Pegoraro E, Plaza C, Romano E, Schädel C, Taylor M, Schuur E (2020) Carbon Thaw Rate Doubles When Accounting for Subsidence in a Permafrost Warming Experiment. J Geophys Res Biogeosciences 125:e2019JG005528. https://doi.org/10.1029/2019JG005528

53. Wang T, Yang D, Yang Y, Piao S, Li X, Cheng G, Fu B (2020) Permafrost thawing puts the frozen carbon at risk over the Tibetan Plateau. Sci Adv 6:eaaz3513. https://doi.org/10.1126/sciadv.aaz3513

54. Magnússon RÍ, Limpens J, Van Huissteden J, Kleijn D, Maximov TC, Rotbarth R, Sass‐Klaassen U, Heijmans MMPD (2020) Rapid Vegetation Succession and Coupled Permafrost Dynamics in Arctic Thaw Ponds in the Siberian Lowland Tundra. J Geophys Res Biogeosciences 125:e2019JG005618. https://doi.org/10.1029/2019JG005618

55. Nitzbon J, Westermann S, Langer M, Martin LCP, Strauss J, Laboor S, Boike J (2020) Fast response of cold ice-rich permafrost in northeast Siberia to a warming climate. Nat Commun 11:2201. https://doi.org/10.1038/s41467-020-15725-8

56. Voigt C, Marushchak ME, Abbott BW, Biasi C, Elberling B, Siciliano SD, Sonnentag O, Stewart KJ, Yang Y, Martikainen PJ (2020) Nitrous oxide emissions from permafrost-affected soils. Nat Rev Earth Environ 1:420–434. https://doi.org/10.1038/s43017-020-0063-9

57. Van Huissteden J (2020) Permafrost in Transition. In: Thawing Permafrost. Springer International Publishing, Cham, pp 275–366

58. Mu C, Abbott BW, Norris AJ, Mu M, Fan C, Chen X, Jia L, Yang R, Zhang T, Wang K, Peng X, Wu Q, Guggenberger G, Wu X (2020) The status and stability of permafrost carbon on the Tibetan Plateau. Earth-Sci Rev 211:103433. https://doi.org/10.1016/j.earscirev.2020.103433

59. Lapham LL, Dallimore SR, Magen C, Henderson LC, Powers LC, Gonsior M, Clark B, Côté M, Fraser P, Orcutt BN (2020) Microbial Greenhouse Gas Dynamics Associated With Warming Coastal Permafrost, Western Canadian Arctic. Front Earth Sci 8:582103. https://doi.org/10.3389/feart.2020.582103

60. Jong D, Bröder L, Tanski G, Fritz M, Lantuit H, Tesi T, Haghipour N, Eglinton TI, Vonk JE (2020) Nearshore Zone Dynamics Determine Pathway of Organic Carbon From Eroding Permafrost Coasts. Geophys Res Lett 47:e2020GL088561. https://doi.org/10.1029/2020GL088561

61. Burke EJ, Zhang Y, Krinner G (2020) Evaluating permafrost physics in the Coupled Model Intercomparison Project 6 (CMIP6) models and their sensitivity to climate change. The Cryosphere 14:3155–3174. https://doi.org/10.5194/tc-14-3155-2020

62. Bröder L, Davydova A, Davydov S, Zimov N, Haghipour N, Eglinton TI, Vonk JE (2020) Particulate Organic Matter Dynamics in a Permafrost Headwater Stream and the Kolyma River Mainstem. J Geophys Res Biogeosciences 125:e2019JG005511. https://doi.org/10.1029/2019JG005511

63. Runge A, Grosse G (2020) Mosaicking Landsat and Sentinel-2 Data to Enhance LandTrendr Time Series Analysis in Northern High Latitude Permafrost Regions. Remote Sens 12:2471. https://doi.org/10.3390/rs12152471

64. Dean JF, Meisel OH, Martyn Rosco M, Marchesini LB, Garnett MH, Lenderink H, Van Logtestijn R, Borges AV, Bouillon S, Lambert T, Röckmann T, Maximov T, Petrov R, Karsanaev S, Aerts R, Van Huissteden J, Vonk JE, Dolman AJ (2020) East Siberian Arctic inland waters emit mostly contemporary carbon. Nat Commun 11:1627. https://doi.org/10.1038/s41467-020-15511-6

65. Jones BM, Tape KD, Clark JA, Nitze I, Grosse G, Disbrow J (2020) Increase in beaver dams controls surface water and thermokarst dynamics in an Arctic tundra region, Baldwin Peninsula, northwestern Alaska. Environ Res Lett 15:075005. https://doi.org/10.1088/1748-9326/ab80f1

66. Wang Y, Xu Y, Wei D, Shi L, Jia Z, Yang Y (2020) Different chemical composition and storage mechanism of soil organic matter between active and permafrost layers on the Qinghai–Tibetan Plateau. J Soils Sediments 20:653–664. https://doi.org/10.1007/s11368-019-02462-9

67. in ’t Zandt MH, Liebner S, Welte CU (2020) Roles of Thermokarst Lakes in a Warming World. Trends Microbiol 28:769–779. https://doi.org/10.1016/j.tim.2020.04.002

68. Macias-Fauria M, Jepson P, Zimov N, Malhi Y (2020) Pleistocene Arctic megafaunal ecological engineering as a natural climate solution? Philos Trans R Soc B Biol Sci 375:20190122. https://doi.org/10.1098/rstb.2019.0122

69. Ahmad D, Hafeez F, Irshad M, Mehmood Q, Tahir AA, Iqbal A, Faridullah (2020) The comparative analysis of essential nutrient fractions in permafrost and different land use systems of Diamer Division, Gilgit-Baltistan. Arab J Geosci 13:1286. https://doi.org/10.1007/s12517-020-06242-5

70. Bhuiyan MAE, Witharana C, Liljedahl AK (2020) Use of Very High Spatial Resolution Commercial Satellite Imagery and Deep Learning to Automatically Map Ice-Wedge Polygons across Tundra Vegetation Types. J Imaging 6:137. https://doi.org/10.3390/jimaging6120137

71. Bengston DN, Crabtree J, Hujala T (2020) Abrupt climate change: Exploring the implications of a wild card. Futures 124:102641. https://doi.org/10.1016/j.futures.2020.102641

72. Marques L (2020) Climate Feedbacks and Tipping Points. In: Capitalism and Environmental Collapse. Springer International Publishing, Cham, pp 199–231

73. Dabrowski JS, Charette MA, Mann PJ, Ludwig SM, Natali SM, Holmes RM, Schade JD, Powell M, Henderson PB (2020) Using radon to quantify groundwater discharge and methane fluxes to a shallow, tundra lake on the Yukon-Kuskokwim Delta, Alaska. Biogeochemistry 148:69–89. https://doi.org/10.1007/s10533-020-00647-w

74. Goudie A (2020) The human impact in geomorphology – 50 years of change. Geomorphology 366:106601. https://doi.org/10.1016/j.geomorph.2018.12.002

75. Meinshausen M, Nicholls ZRJ, Lewis J, Gidden MJ, Vogel E, Freund M, Beyerle U, Gessner C, Nauels A, Bauer N, Canadell JG, Daniel JS, John A, Krummel PB, Luderer G, Meinshausen N, Montzka SA, Rayner PJ, Reimann S, Smith SJ, Van Den Berg M, Velders GJM, Vollmer MK, Wang RHJ (2020) The shared socio-economic pathway (SSP) greenhouse gas concentrations and their extensions to 2500. Geosci Model Dev 13:3571–3605. https://doi.org/10.5194/gmd-13-3571-2020

76. Hugelius G, Loisel J, Chadburn S, Jackson RB, Jones M, MacDonald G, Marushchak M, Olefeldt D, Packalen M, Siewert MB, Treat C, Turetsky M, Voigt C, Yu Z (2020) Large stocks of peatland carbon and nitrogen are vulnerable to permafrost thaw. Proc Natl Acad Sci 117:20438–20446. https://doi.org/10.1073/pnas.1916387117

77. Tank SE, Vonk JE, Walvoord MA, McClelland JW, Laurion I, Abbott BW (2020) Landscape matters: Predicting the biogeochemical effects of permafrost thaw on aquatic networks with a state factor approach. Permafr Periglac Process 31:358–370. https://doi.org/10.1002/ppp.2057

78. Gagné KR, Ewers SC, Murphy CJ, Daanen R, Walter Anthony K, Guerard JJ (2020) Composition and photo-reactivity of organic matter from permafrost soils and surface waters in interior Alaska. Environ Sci Process Impacts 22:1525–1539. https://doi.org/10.1039/D0EM00097C

79. Knoblauch C, Beer C, Schuett A, Sauerland L, Liebner S, Steinhof A, Rethemeyer J, Grigoriev MN, Faguet A, Pfeiffer E (2021) Carbon Dioxide and Methane Release Following Abrupt Thaw of Pleistocene Permafrost Deposits in Arctic Siberia. J Geophys Res Biogeosciences 126:. https://doi.org/10.1029/2021JG006543

80. Natali SM, Holdren JP, Rogers BM, Treharne R, Duffy PB, Pomerance R, MacDonald E (2021) Permafrost carbon feedbacks threaten global climate goals. Proc Natl Acad Sci 118:e2100163118. https://doi.org/10.1073/pnas.2100163118

81. Walter Anthony KM, Lindgren P, Hanke P, Engram M, Anthony P, Daanen RP, Bondurant A, Liljedahl AK, Lenz J, Grosse G, Jones BM, Brosius L, James SR, Minsley BJ, Pastick NJ, Munk J, Chanton JP, Miller CE, Meyer FJ (2021) Decadal-scale hotspot methane ebullition within lakes following abrupt permafrost thaw. Environ Res Lett 16:035010. https://doi.org/10.1088/1748-9326/abc848

82. Gibson CM, Brinkman T, Cold H, Brown D, Turetsky M (2021) Identifying increasing risks of hazards for northern land-users caused by permafrost thaw: integrating scientific and community-based research approaches. Environ Res Lett 16:064047. https://doi.org/10.1088/1748-9326/abfc79

83. Anisimov O, Zimov S (2021) Thawing permafrost and methane emission in Siberia: Synthesis of observations, reanalysis, and predictive modeling. Ambio 50:2050–2059. https://doi.org/10.1007/s13280-020-01392-y

84. Elder CD, Thompson DR, Thorpe AK, Chandanpurkar HA, Hanke PJ, Hasson N, James SR, Minsley BJ, Pastick NJ, Olefeldt D, Walter Anthony KM, Miller CE (2021) Characterizing Methane Emission Hotspots From Thawing Permafrost. Glob Biogeochem Cycles 35:e2020GB006922. https://doi.org/10.1029/2020GB006922

85. Jin X-Y, Jin H-J, Iwahana G, Marchenko SS, Luo D-L, Li X-Y, Liang S-H (2021) Impacts of climate-induced permafrost degradation on vegetation: A review. Adv Clim Change Res 12:29–47. https://doi.org/10.1016/j.accre.2020.07.002

86. Treat CC, Jones MC, Alder J, Sannel ABK, Camill P, Frolking S (2021) Predicted Vulnerability of Carbon in Permafrost Peatlands With Future Climate Change and Permafrost Thaw in Western Canada. J Geophys Res Biogeosciences 126:e2020JG005872. https://doi.org/10.1029/2020JG005872

87. Miner KR, D’Andrilli J, Mackelprang R, Edwards A, Malaska MJ, Waldrop MP, Miller CE (2021) Emergent biogeochemical risks from Arctic permafrost degradation. Nat Clim Change 11:809–819. https://doi.org/10.1038/s41558-021-01162-y

88. Waldrop MP, W. McFarland J, Manies KL, Leewis MC, Blazewicz SJ, Jones MC, Neumann RB, Keller JK, Cohen L, Euskirchen ES, Edgar C, Turetsky MR, Cable WL (2021) Carbon Fluxes and Microbial Activities From Boreal Peatlands Experiencing Permafrost Thaw. J Geophys Res Biogeosciences 126:e2020JG005869. https://doi.org/10.1029/2020JG005869

89. Kuhn MA, Thompson LM, Winder JC, Braga LPP, Tanentzap AJ, Bastviken D, Olefeldt D (2021) Opposing Effects of Climate and Permafrost Thaw on CH _4_ and CO _2_ Emissions From Northern Lakes. AGU Adv 2:e2021AV000515. https://doi.org/10.1029/2021AV000515

90. Marushchak ME, Kerttula J, Diáková K, Faguet A, Gil J, Grosse G, Knoblauch C, Lashchinskiy N, Martikainen PJ, Morgenstern A, Nykamb M, Ronkainen JG, Siljanen HMP, Van Delden L, Voigt C, Zimov N, Zimov S, Biasi C (2021) Thawing Yedoma permafrost is a neglected nitrous oxide source. Nat Commun 12:7107. https://doi.org/10.1038/s41467-021-27386-2

91. Bröder L, Keskitalo K, Zolkos S, Shakil S, Tank SE, Kokelj SV, Tesi T, Van Dongen BE, Haghipour N, Eglinton TI, Vonk JE (2021) Preferential export of permafrost-derived organic matter as retrogressive thaw slumping intensifies. Environ Res Lett 16:054059. https://doi.org/10.1088/1748-9326/abee4b

92. Strauss J, Laboor S, Schirrmeister L, Fedorov AN, Fortier D, Froese D, Fuchs M, Günther F, Grigoriev M, Harden J, Hugelius G, Jongejans LL, Kanevskiy M, Kholodov A, Kunitsky V, Kraev G, Lozhkin A, Rivkina E, Shur Y, Siegert C, Spektor V, Streletskaya I, Ulrich M, Vartanyan S, Veremeeva A, Anthony KW, Wetterich S, Zimov N, Grosse G (2021) Circum-Arctic Map of the Yedoma Permafrost Domain. Front Earth Sci 9:758360. https://doi.org/10.3389/feart.2021.758360

93. Keskitalo KH, Bröder L, Shakil S, Zolkos S, Tank SE, Van Dongen BE, Tesi T, Haghipour N, Eglinton TI, Kokelj SV, Vonk JE (2021) Downstream Evolution of Particulate Organic Matter Composition From Permafrost Thaw Slumps. Front Earth Sci 9:642675. https://doi.org/10.3389/feart.2021.642675

94. MacDougall AH (2021) Estimated effect of the permafrost carbon feedback on the zero emissions commitment to climate change. Biogeosciences 18:4937–4952. https://doi.org/10.5194/bg-18-4937-2021

95. Gibson C, Cottenie K, Gingras-Hill T, Kokelj SV, Baltzer JL, Chasmer L, Turetsky MR (2021) Mapping and understanding the vulnerability of northern peatlands to permafrost thaw at scales relevant to community adaptation planning. Environ Res Lett 16:055022. https://doi.org/10.1088/1748-9326/abe74b

96. Siewert MB, Lantuit H, Richter A, Hugelius G (2021) Permafrost Causes Unique Fine‐Scale Spatial Variability Across Tundra Soils. Glob Biogeochem Cycles 35:e2020GB006659. https://doi.org/10.1029/2020GB006659

97. Chen Y, Romps DM, Seeley JT, Veraverbeke S, Riley WJ, Mekonnen ZA, Randerson JT (2021) Future increases in Arctic lightning and fire risk for permafrost carbon. Nat Clim Change 11:404–410. https://doi.org/10.1038/s41558-021-01011-y

98. Woodard DL, Shiklomanov AN, Kravitz B, Hartin C, Bond-Lamberty B (2021) A permafrost implementation in the simple carbon–climate model Hector v.2.3pf. Geosci Model Dev 14:4751–4767. https://doi.org/10.5194/gmd-14-4751-2021

99. Rogers JA, Galy V, Kellerman AM, Chanton JP, Zimov N, Spencer RGM (2021) Limited Presence of Permafrost Dissolved Organic Matter in the Kolyma River, Siberia Revealed by Ramped Oxidation. J Geophys Res Biogeosciences 126:e2020JG005977. https://doi.org/10.1029/2020JG005977

100. Jones BM, Tape KD, Clark JA, Bondurant AC, Ward Jones MK, Gaglioti BV, Elder CD, Witharana C, Miller CE (2021) Multi-Dimensional Remote Sensing Analysis Documents Beaver-Induced Permafrost Degradation, Seward Peninsula, Alaska. Remote Sens 13:4863. https://doi.org/10.3390/rs13234863

101. Wologo E, Shakil S, Zolkos S, Textor S, Ewing S, Klassen J, Spencer RGM, Podgorski DC, Tank SE, Baker MA, O’Donnell JA, Wickland KP, Foks SSW, Zarnetske JP, Lee‐Cullin J, Liu F, Yang Y, Kortelainen P, Kolehmainen J, Dean JF, Vonk JE, Holmes RM, Pinay G, Powell MM, Howe J, Frei RJ, Bratsman SP, Abbott BW (2021) Stream Dissolved Organic Matter in Permafrost Regions Shows Surprising Compositional Similarities but Negative Priming and Nutrient Effects. Glob Biogeochem Cycles 35:e2020GB006719. https://doi.org/10.1029/2020GB006719

102. Abbott BW, Rocha AV, Shogren A, Zarnetske JP, Iannucci F, Bowden WB, Bratsman SP, Patch L, Watts R, Fulweber R, Frei RJ, Huebner AM, Ludwig SM, Carling GT, O’Donnell JA (2021) Tundra wildfire triggers sustained lateral nutrient loss in Alaskan Arctic. Glob Change Biol 27:1408–1430. https://doi.org/10.1111/gcb.15507

103. Magnússon RÍ, Limpens J, Kleijn D, Van Huissteden K, Maximov TC, Lobry S, Heijmans MMPD (2021) Shrub decline and expansion of wetland vegetation revealed by very high resolution land cover change detection in the Siberian lowland tundra. Sci Total Environ 782:146877. https://doi.org/10.1016/j.scitotenv.2021.146877

104. Christensen TR, Lund M, Skov K, Abermann J, López-Blanco E, Scheller J, Scheel M, Jackowicz-Korczynski M, Langley K, Murphy MJ, Mastepanov M (2021) Multiple Ecosystem Effects of Extreme Weather Events in the Arctic. Ecosystems 24:122–136. https://doi.org/10.1007/s10021-020-00507-6

105. Pfeiffer EM, Vybornova O 77Kutzbach, Fedorova I, Knoblauch C, Tsibizov L, Beer C (2021) Focus Siberian Permafrost Terrestrial Cryosphere and Climate Change. Alfred-Wegener-Institut, Helmholtz-Zentrum für Polar- und Meeresforschung

106. Behnke MI, McClelland JW, Tank SE, Kellerman AM, Holmes RM, Haghipour N, Eglinton TI, Raymond PA, Suslova A, Zhulidov AV, Gurtovaya T, Zimov N, Zimov S, Mutter EA, Amos E, Spencer RGM (2021) Pan‐Arctic Riverine Dissolved Organic Matter: Synchronous Molecular Stability, Shifting Sources and Subsidies. Glob Biogeochem Cycles 35:e2020GB006871. https://doi.org/10.1029/2020GB006871

107. Jongejans LL, Liebner S, Knoblauch C, Mangelsdorf K, Ulrich M, Grosse G, Tanski G, Fedorov AN, Konstantinov PYa, Windirsch T, Wiedmann J, Strauss J (2021) Greenhouse gas production and lipid biomarker distribution in Yedoma and Alas thermokarst lake sediments in Eastern Siberia. Glob Change Biol 27:2822–2839. https://doi.org/10.1111/gcb.15566

108. Monhonval A, Mauclet E, Pereira B, Vandeuren A, Strauss J, Grosse G, Schirrmeister L, Fuchs M, Kuhry P, Opfergelt S (2021) Mineral Element Stocks in the Yedoma Domain: A Novel Method Applied to Ice-Rich Permafrost Regions. Front Earth Sci 9:703304. https://doi.org/10.3389/feart.2021.703304

109. Yin G, Luo J, Niu F, Lin Z, Liu M (2021) Machine learning-based thermokarst landslide susceptibility modeling across the permafrost region on the Qinghai-Tibet Plateau. Landslides 18:2639–2649. https://doi.org/10.1007/s10346-021-01669-7

110. Bruhwiler L, Parmentier F-JW, Crill P, Leonard M, Palmer PI (2021) The Arctic Carbon Cycle and Its Response to Changing Climate. Curr Clim Change Rep 7:14–34. https://doi.org/10.1007/s40641-020-00169-5

111. Philipp M, Dietz A, Buchelt S, Kuenzer C (2021) Trends in Satellite Earth Observation for Permafrost Related Analyses—A Review. Remote Sens 13:1217. https://doi.org/10.3390/rs13061217

112. Andresen CG, Lougheed VL (2021) Arctic aquatic graminoid tundra responses to nutrient availability. Biogeosciences 18:2649–2662. https://doi.org/10.5194/bg-18-2649-2021

113. Liu C, Huang H, Sun F (2021) A Pixel-Based Vegetation Greenness Trend Analysis over the Russian Tundra with All Available Landsat Data from 1984 to 2018. Remote Sens 13:4933. https://doi.org/10.3390/rs13234933

114. Slaymaker O, Spencer T, Embleton-Hamann C (2021) Recasting geomorphology as a landscape science. Geomorphology 384:107723. https://doi.org/10.1016/j.geomorph.2021.107723

115. Van Huissteden J, Teshebaeva K, Cheung Y, Magnússon RÍ, Noorbergen H, Karsanaev SV, Maximov TC, Dolman AJ (2021) Geomorphology and InSAR-Tracked Surface Displacements in an Ice-Rich Yedoma Landscape. Front Earth Sci 9:680565. https://doi.org/10.3389/feart.2021.680565

116. Jandt RR (Randi R), Miller EA, Jones BN (2021) Fire effects 10 years after the Anaktuvuk River Tundra fire. Anchorage, Alaska, U.S. Department of the Interior, Bureau of Land Management, 2021

117. Scheller JH, Mastepanov M, Christiansen HH, Christensen TR (2021) Methane in Zackenberg Valley, NE Greenland: multidecadal growing season fluxes of a high-Arctic tundra. Biogeosciences 18:6093–6114. https://doi.org/10.5194/bg-18-6093-2021

118. Miner KR, Turetsky MR, Malina E, Bartsch A, Tamminen J, McGuire AD, Fix A, Sweeney C, Elder CD, Miller CE (2022) Permafrost carbon emissions in a changing Arctic. Nat Rev Earth Environ 3:55–67. https://doi.org/10.1038/s43017-021-00230-3

119. Rodenhizer H, Belshe F, Celis G, Ledman J, Mauritz M, Goetz S, Sankey T, Schuur EAG (2022) Abrupt permafrost thaw accelerates carbon dioxide and methane release at a tussock tundra site. Arct Antarct Alp Res 54:443–464. https://doi.org/10.1080/15230430.2022.2118639

120. Heijmans MMPD, Magnússon RÍ, Lara MJ, Frost GV, Myers-Smith IH, van Huissteden J, Jorgenson MT, Fedorov AN, Epstein HE, Lawrence DM, Limpens J (2022) Tundra vegetation change and impacts on permafrost. Nat Rev Earth Environ 3:68–84. https://doi.org/10.1038/s43017-021-00233-0

121. Runge A, Nitze I, Grosse G (2022) Remote sensing annual dynamics of rapid permafrost thaw disturbances with LandTrendr. Remote Sens Environ 268:112752. https://doi.org/10.1016/j.rse.2021.112752

122. Webb EE, Liljedahl AK, Cordeiro JA, Loranty MM, Witharana C, Lichstein JW (2022) Permafrost thaw drives surface water decline across lake-rich regions of the Arctic. Nat Clim Change 12:841–846. https://doi.org/10.1038/s41558-022-01455-w

123. Ernakovich JG, Barbato RA, Rich VI, Schädel C, Hewitt RE, Doherty SJ, Whalen ED, Abbott BW, Barta J, Biasi C, Chabot CL, Hultman J, Knoblauch C, Vetter MCYL, Leewis M, Liebner S, Mackelprang R, Onstott TC, Richter A, Schütte UME, Siljanen HMP, Taş N, Timling I, Vishnivetskaya TA, Waldrop MP, Winkel M (2022) Microbiome assembly in thawing permafrost and its feedbacks to climate. Glob Change Biol 28:5007–5026. https://doi.org/10.1111/gcb.16231

124. Pellerin A, Lotem N, Walter Anthony K, Eliani Russak E, Hasson N, Røy H, Chanton JP, Sivan O (2022) Methane production controls in a young thermokarst lake formed by abrupt permafrost thaw. Glob Change Biol 28:3206–3221. https://doi.org/10.1111/gcb.16151

125. Minsley BJ, Pastick NJ, James SR, Brown DRN, Wylie BK, Kass MA, Romanovsky VE (2022) Rapid and Gradual Permafrost Thaw: A Tale of Two Sites. Geophys Res Lett 49:e2022GL100285. https://doi.org/10.1029/2022GL100285

126. Hjort J, Streletskiy D, Doré G, Wu Q, Bjella K, Luoto M (2022) Impacts of permafrost degradation on infrastructure. Nat Rev Earth Environ 3:24–38. https://doi.org/10.1038/s43017-021-00247-8

127. Scheel M, Zervas A, Jacobsen CS, Christensen TR (2022) Microbial Community Changes in 26,500-Year-Old Thawing Permafrost. Front Microbiol 13:787146. https://doi.org/10.3389/fmicb.2022.787146

128. Wegner R, Fiencke C, Knoblauch C, Sauerland L, Beer C (2022) Rapid Permafrost Thaw Removes Nitrogen Limitation and Rises the Potential for N2O Emissions. Nitrogen 3:608–627. https://doi.org/10.3390/nitrogen3040040

129. Burke E, Chadburn S, Huntingford C (2022) Thawing Permafrost as a Nitrogen Fertiliser: Implications for Climate Feedbacks. Nitrogen 3:353–375. https://doi.org/10.3390/nitrogen3020023

130. Strauss J, Biasi C, Sanders T, Abbott BW, von Deimling TS, Voigt C, Winkel M, Marushchak ME, Kou D, Fuchs M, Horn MA, Jongejans LL, Liebner S, Nitzbon J, Schirrmeister L, Walter Anthony K, Yang Y, Zubrzycki S, Laboor S, Treat C, Grosse G (2022) A globally relevant stock of soil nitrogen in the Yedoma permafrost domain. Nat Commun 13:6074. https://doi.org/10.1038/s41467-022-33794-9

131. Schuur EAG, Abbott BW, Commane R, Ernakovich J, Euskirchen E, Hugelius G, Grosse G, Jones M, Koven C, Leshyk V, Lawrence D, Loranty MM, Mauritz M, Olefeldt D, Natali S, Rodenhizer H, Salmon V, Schädel C, Strauss J, Treat C, Turetsky M (2022) Permafrost and Climate Change: Carbon Cycle Feedbacks From the Warming Arctic. Annu Rev Environ Resour 47:343–371. https://doi.org/10.1146/annurev-environ-012220-011847

132. Chen Y, Liu A, Cheng X (2022) Landsat-Based Monitoring of Landscape Dynamics in Arctic Permafrost Region. J Remote Sens 2022:2022/9765087. https://doi.org/10.34133/2022/9765087

133. Treharne R, Rogers BM, Gasser T, MacDonald E, Natali S (2022) Identifying Barriers to Estimating Carbon Release From Interacting Feedbacks in a Warming Arctic. Front Clim 3:716464. https://doi.org/10.3389/fclim.2021.716464

134. Abbott BW (2022) Permafrost Climate Feedbacks. In: Finger M, Rekvig G (eds) Global Arctic. Springer International Publishing, Cham, pp 189–209

135. Hirst C, Mauclet E, Monhonval A, Tihon E, Ledman J, Schuur EAG, Opfergelt S (2022) Seasonal Changes in Hydrology and Permafrost Degradation Control Mineral Element‐Bound DOC Transport From Permafrost Soils to Streams. Glob Biogeochem Cycles 36:e2021GB007105. https://doi.org/10.1029/2021GB007105

136. Chen Y, Liu A, Cheng X (2022) Detection of thermokarst lake drainage events in the northern Alaska permafrost region. Sci Total Environ 807:150828. https://doi.org/10.1016/j.scitotenv.2021.150828

137. Wang P, Huang Q, Liu S, Cai H, Yu J, Wang T, Chen X, Pozdniakov SP (2022) Recent regional warming across the Siberian lowlands: a comparison between permafrost and non-permafrost areas. Environ Res Lett 17:054047. https://doi.org/10.1088/1748-9326/ac6c9d

138. Patzner MS, Logan M, McKenna AM, Young RB, Zhou Z, Joss H, Mueller CW, Hoeschen C, Scholten T, Straub D, Kleindienst S, Borch T, Kappler A, Bryce C (2022) Microbial iron cycling during palsa hillslope collapse promotes greenhouse gas emissions before complete permafrost thaw. Commun Earth Environ 3:76. https://doi.org/10.1038/s43247-022-00407-8

139. Bernhard P, Zwieback S, Hajnsek I (2022) Accelerated mobilization of organic carbon from retrogressive thaw slumps on the northern Taymyr Peninsula. The Cryosphere 16:2819–2835. https://doi.org/10.5194/tc-16-2819-2022

140. Treat CC, Jones MC, Alder J, Frolking S (2022) Hydrologic Controls on Peat Permafrost and Carbon Processes: New Insights From Past and Future Modeling. Front Environ Sci 10:892925. https://doi.org/10.3389/fenvs.2022.892925

141. Wei Z, Du Z, Wang L, Zhong W, Lin J, Xu Q, Xiao C (2022) Sedimentary organic carbon storage of thermokarst lakes and ponds across Tibetan permafrost region. Sci Total Environ 831:154761. https://doi.org/10.1016/j.scitotenv.2022.154761

142. Witharana C, Udawalpola MR, Liljedahl AK, Jones MKW, Jones BM, Hasan A, Joshi D, Manos E (2022) Automated Detection of Retrogressive Thaw Slumps in the High Arctic Using High-Resolution Satellite Imagery. Remote Sens 14:4132. https://doi.org/10.3390/rs14174132

143. Smith ND, Burke EJ, Schanke Aas K, Althuizen IHJ, Boike J, Christiansen CT, Etzelmüller B, Friborg T, Lee H, Rumbold H, Turton RH, Westermann S, Chadburn SE (2022) Explicitly modelling microtopography in permafrost landscapes in a land surface model (JULES vn5.4_microtopography). Geosci Model Dev 15:3603–3639. https://doi.org/10.5194/gmd-15-3603-2022

144. Natali SM, Bronen R, Cochran P, Holdren JP, Rogers BM, Treharne R (2022) Incorporating permafrost into climate mitigation and adaptation policy. Environ Res Lett 17:091001. https://doi.org/10.1088/1748-9326/ac8c5a

145. Fiencke C, Marushchak ME, Sanders T, Wegner R, Beer C (2022) Microbiogeochemical Traits to Identify Nitrogen Hotspots in Permafrost Regions. Nitrogen 3:458–501. https://doi.org/10.3390/nitrogen3030031

146. Korosi JB, Coleman KA, Hoskin GN, Little AJ, Stewart EM, Thienpont JR (2022) Paleolimnological perspectives on the shifting geographic template of permafrost landscapes and its implications for Arctic freshwater biodiversity. Can J Fish Aquat Sci 79:1162–1172. https://doi.org/10.1139/cjfas-2021-0280

147. Freitas P, Vieira G, Mora C, Canário J, Vincent WF (2022) Vegetation shadow casts impact remotely sensed reflectance from permafrost thaw ponds in the subarctic forest-tundra zone. Environ Earth Sci 81:522. https://doi.org/10.1007/s12665-022-10640-1

148. Jongejans LL, Mangelsdorf K, Karger C, Opel T, Wetterich S, Courtin J, Meyer H, Kizyakov AI, Grosse G, Shepelev AG, Syromyatnikov II, Fedorov AN, Strauss J (2022) Molecular biomarkers in Batagay megaslump permafrost deposits reveal clear differences in organic matter preservation between glacial and interglacial periods. The Cryosphere 16:3601–3617. https://doi.org/10.5194/tc-16-3601-2022

149. Shakil S, Tank SE, Vonk JE, Zolkos S (2022) Low biodegradability of particulate organic carbon mobilized from thaw slumps on the Peel Plateau, NT, and possible chemosynthesis and sorption effects. Biogeosciences 19:1871–1890. https://doi.org/10.5194/bg-19-1871-2022

150. Tape KD, Clark JA, Jones BM, Kantner S, Gaglioti BV, Grosse G, Nitze I (2022) Expanding beaver pond distribution in Arctic Alaska, 1949 to 2019. Sci Rep 12:7123. https://doi.org/10.1038/s41598-022-09330-6

151. Del Vecchio J, DiBiase RA, Corbett LB, Bierman PR, Caffee MW, Ivory SJ (2022) Increased Erosion Rates Following the Onset of Pleistocene Periglaciation at Bear Meadows, Pennsylvania, USA. Geophys Res Lett 49:e2021GL096739. https://doi.org/10.1029/2021GL096739

152. Keskitalo KH, Bröder L, Jong D, Zimov N, Davydova A, Davydov S, Tesi T, Mann PJ, Haghipour N, Eglinton TI, Vonk JE (2022) Seasonal variability in particulate organic carbon degradation in the Kolyma River, Siberia. Environ Res Lett 17:034007. https://doi.org/10.1088/1748-9326/ac4f8d

153. Baskaran L, Elder C, Bloom AA, Ma S, Thompson D, Miller CE (2022) Geomorphological patterns of remotely sensed methane hot spots in the Mackenzie Delta, Canada. Environ Res Lett 17:015009. https://doi.org/10.1088/1748-9326/ac41fb

154. Armstrong McKay DI, Staal A, Abrams JF, Winkelmann R, Sakschewski B, Loriani S, Fetzer I, Cornell SE, Rockström J, Lenton TM (2022) Exceeding 1.5°C global warming could trigger multiple climate tipping points. Science 377:eabn7950. https://doi.org/10.1126/science.abn7950

155. Kuhry P, Makopoulou E, Pascual Descarrega D, Pecker Marcosig I, Trombotto Liaudat D (2022) Soil organic carbon stocks in the high mountain permafrost zone of the semi-arid Central Andes (Cordillera Frontal, Argentina). CATENA 217:106434. https://doi.org/10.1016/j.catena.2022.106434

156. Van Oort B, Lund MT, Brisebois A (2022) Climate Change in Northern Regions. In: Tryland M (ed) Arctic One Health. Springer International Publishing, Cham, pp 79–119

157. Cuesta-Valero FJ, Beltrami H, García-García A, Krinner G, Langer M, MacDougall AH, Nitzbon J, Peng J, Von Schuckmann K, Seneviratne SI, Thiery W, Vanderkelen I, Wu T (2023) Continental heat storage: contributions from the ground, inland waters, and permafrost thawing. Earth Syst Dyn 14:609–627. https://doi.org/10.5194/esd-14-609-2023

158. Rodenhizer H, Natali SM, Mauritz M, Taylor MA, Celis G, Kadej S, Kelley AK, Lathrop ER, Ledman J, Pegoraro EF, Salmon VG, Schädel C, See C, Webb EE, Schuur EAG (2023) Abrupt permafrost thaw drives spatially heterogeneous soil moisture and carbon dioxide fluxes in upland tundra. Glob Change Biol 29:6286–6302. https://doi.org/10.1111/gcb.16936

159. Painter SL, Coon ET, Khattak AJ, Jastrow JD (2023) Drying of tundra landscapes will limit subsidence-induced acceleration of permafrost thaw. Proc Natl Acad Sci 120:e2212171120. https://doi.org/10.1073/pnas.2212171120

160. Scheel M, Zervas A, Rijkers R, Tveit AT, Ekelund F, Campuzano Jiménez F, Christensen TR, Jacobsen CS (2023) Abrupt permafrost thaw triggers activity of copiotrophs and microbiome predators. FEMS Microbiol Ecol 99:fiad123. https://doi.org/10.1093/femsec/fiad123

161. Bartsch A, Strozzi T, Nitze I (2023) Permafrost Monitoring from Space. Surv Geophys 44:1579–1613. https://doi.org/10.1007/s10712-023-09770-3

162. Jones K, Berggren M, Sjöstedt J (2023) Seasonal variation and importance of catchment area composition for transport of bioavailable carbon to the Baltic Sea. Biogeochemistry 165:265–276. https://doi.org/10.1007/s10533-023-01079-y

163. Mu M, Mu C, Liu H, Chi H, Zhu Y, Shang J, Fan C, Wu X, Zhang G (2023) Carbon loss and emissions within a permafrost collapse chronosequence. CATENA 231:107291. https://doi.org/10.1016/j.catena.2023.107291

164. Monhonval A, Hirst C, Strauss J, Schuur EAG, Opfergelt S (2023) Strontium isotopes trace the dissolution and precipitation of mineral organic carbon interactions in thawing permafrost. Geoderma 433:116456. https://doi.org/10.1016/j.geoderma.2023.116456

165. Miner KR, Hollis JR, Miller CE, Uckert K, Douglas TA, Cardarelli E, Mackelprang R (2023) Earth to Mars: A Protocol for Characterizing Permafrost in the Context of Climate Change as an Analog for Extraplanetary Exploration. Astrobiology 23:1006–1018. https://doi.org/10.1089/ast.2022.0155

166. Gay BA, Pastick NJ, Züfle AE, Armstrong AH, Miner KR, Qu JJ (2023) Investigating permafrost carbon dynamics in Alaska with artificial intelligence. Environ Res Lett 18:125001. https://doi.org/10.1088/1748-9326/ad0607

167. Beer C, Runge A, Grosse G, Hugelius G, Knoblauch C (2023) Carbon dioxide release from retrogressive thaw slumps in Siberia. Environ Res Lett 18:104053. https://doi.org/10.1088/1748-9326/acfdbb

168. Thomas M, Monhonval A, Hirst C, Bröder L, Zolkos S, Vonk JE, Tank SE, Keskitalo KH, Shakil S, Kokelj SV, Van Der Sluijs J, Opfergelt S (2023) Evidence for preservation of organic carbon interacting with iron in material displaced from retrogressive thaw slumps: Case study in Peel Plateau, western Canadian Arctic. Geoderma 433:116443. https://doi.org/10.1016/j.geoderma.2023.116443

169. Murton J, Opel T, Wetterich S, Ashastina K, Savvinov G, Danilov P, Boeskorov V (2023) Batagay megaslump: A review of the permafrost deposits, Quaternary environmental history, and recent development. Permafr Periglac Process 34:399–416. https://doi.org/10.1002/ppp.2194

170. Kemeny PC, Li GK, Douglas M, Berelson W, Chadwick AJ, Dalleska NF, Lamb MP, Larsen W, Magyar JS, Rollins NE, Rowland J, Smith MI, Torres MA, Webb SM, Fischer WW, West AJ (2023) Arctic Permafrost Thawing Enhances Sulfide Oxidation. Glob Biogeochem Cycles 37:e2022GB007644. https://doi.org/10.1029/2022GB007644

171. Yang G, Zheng Z, Abbott BW, Olefeldt D, Knoblauch C, Song Y, Kang L, Qin S, Peng Y, Yang Y (2023) Characteristics of methane emissions from alpine thermokarst lakes on the Tibetan Plateau. Nat Commun 14:3121. https://doi.org/10.1038/s41467-023-38907-6

172. Jiao Z, Xu Z, Guo R, Zhou Z, Jiang L (2023) Potential of Multi-temporal InSAR for Detecting Retrogressive Thaw Slumps: A Case of the Beiluhe Region of the Tibetan Plateau. Int J Disaster Risk Sci 14:523–538. https://doi.org/10.1007/s13753-023-00505-x

173. Verfaillie M, Cho E, Dwyre L, Khan I, Wagner C, Jacobs JM, Hunsaker A (2023) UAS remote sensing applications to abrupt cold region hazards. Front Remote Sens 4:1095275. https://doi.org/10.3389/frsen.2023.1095275

174. Brosius LS, Walter Anthony KM, Treat CC, Jones MC, Dyonisius M, Grosse G (2023) Panarctic lakes exerted a small positive feedback on early Holocene warming due to deglacial release of methane. Commun Earth Environ 4:271. https://doi.org/10.1038/s43247-023-00930-2

175. Arndt KA, Hashemi J, Natali SM, Schiferl LD, Virkkala A-M (2023) Recent Advances and Challenges in Monitoring and Modeling Non-Growing Season Carbon Dioxide Fluxes from the Arctic Boreal Zone. Curr Clim Change Rep 9:27–40. https://doi.org/10.1007/s40641-023-00190-4

176. Wang S, Foster A, Lenz EA, Kessler JD, Stroeve JC, Anderson LO, Turetsky M, Betts R, Zou S, Liu W, Boos WR, Hausfather Z (2023) Mechanisms and Impacts of Earth System Tipping Elements. Rev Geophys 61:e2021RG000757. https://doi.org/10.1029/2021RG000757

177. Wagner J, Martin V, Speetjens NJ, A’Campo W, Durstewitz L, Lodi R, Fritz M, Tanski G, Vonk JE, Richter A, Bartsch A, Lantuit H, Hugelius G (2023) High resolution mapping shows differences in soil carbon and nitrogen stocks in areas of varying landscape history in Canadian lowland tundra. Geoderma 438:116652. https://doi.org/10.1016/j.geoderma.2023.116652

178. Sjöberg Y, Bouchard F, Gartler S, Bartsch A, Zona D (2023) Focus on Arctic change: transdisciplinary research and communication. Environ Res Lett 18:010201. https://doi.org/10.1088/1748-9326/acabd7

179. Winkelmann R, Steinert D, McKay A, Brovkin V, Kääb A, Notz D, Aksenov Y, Arndt S, Bathiany S, Burke E, Garbe J, Gasson E, Goelzer H, Hugelius G, Klose AK, Langebroek P, Marzeion B, Maussion F, Nitzbon J, Robinson A, Rynders S, Sudakow I (2023) Global Tipping Points Report 2023: Ch 1.2: Cryosphere tipping points. University of Exeter

180. Palazzo Corner S, Siegert M, Ceppi P, Fox-Kemper B, Frölicher TL, Gallego-Sala A, Haigh J, Hegerl GC, Jones CD, Knutti R, Koven CD, MacDougall AH, Meinshausen M, Nicholls Z, Sallée JB, Sanderson BM, Séférian R, Turetsky M, Williams RG, Zaehle S, Rogelj J (2023) The Zero Emissions Commitment and climate stabilization. Front Sci 1:1170744. https://doi.org/10.3389/fsci.2023.1170744

181. Shannon KC, Christman NR, Crump BC, Carey MP, Koch J, Lapham LL, O’Donnell J, Poulin BA, Tape KD, Clark JA, Colwell FS (2023) Comparing Sediment Microbial Communities of Arctic Beaver Ponds to Tundra Lakes and Streams. J Geophys Res Biogeosciences 128:e2023JG007408. https://doi.org/10.1029/2023JG007408

182. Saros JE, Arp CD, Bouchard F, Comte J, Couture R-M, Dean JF, Lafrenière M, MacIntyre S, McGowan S, Rautio M, Prater C, Tank SE, Walvoord M, Wickland KP, Antoniades D, Ayala-Borda P, Canario J, Drake TW, Folhas D, Hazuková V, Kivilä H, Klanten Y, Lamoureux S, Laurion I, Pilla RM, Vonk JE, Zolkos S, Vincent WF (2023) Sentinel responses of Arctic freshwater systems to climate: linkages, evidence, and a roadmap for future research. Arct Sci 9:356–392. https://doi.org/10.1139/as-2022-0021

183. Hessen DO, Andersen T, Armstrong McKay D, Kosten S, Meerhoff M, Pickard A, Spears BM (2024) Lake ecosystem tipping points and climate feedbacks. Earth Syst Dyn 15:653–669. https://doi.org/10.5194/esd-15-653-2024

184. Nitzbon J, Schneider Von Deimling T, Aliyeva M, Chadburn SE, Grosse G, Laboor S, Lee H, Lohmann G, Steinert NJ, Stuenzi SM, Werner M, Westermann S, Langer M (2024) No respite from permafrost-thaw impacts in the absence of a global tipping point. Nat Clim Change 14:573–585. https://doi.org/10.1038/s41558-024-02011-4

185. Parmentier FW, Nilsen L, Tømmervik H, Meisel OH, Bröder L, Vonk JE, Westermann S, Semenchuk PR, Cooper EJ (2024) Rapid Ice‐Wedge Collapse and Permafrost Carbon Loss Triggered by Increased Snow Depth and Surface Runoff. Geophys Res Lett 51:e2023GL108020. https://doi.org/10.1029/2023GL108020

186. Ramage J, Kuhn M, Virkkala A, Voigt C, Marushchak ME, Bastos A, Biasi C, Canadell JG, Ciais P, López‐Blanco E, Natali SM, Olefeldt D, Potter S, Poulter B, Rogers BM, Schuur EAG, Treat C, Turetsky MR, Watts J, Hugelius G (2024) The Net GHG Balance and Budget of the Permafrost Region (2000–2020) From Ecosystem Flux Upscaling. Glob Biogeochem Cycles 38:e2023GB007953. https://doi.org/10.1029/2023GB007953

187. Zhu X, Jia G, Xu X (2024) Accelerated rise in wildfire carbon emissions from Arctic continuous permafrost. Sci Bull S2095927324003566. https://doi.org/10.1016/j.scib.2024.05.022

188. Treat CC, Virkkala A, Burke E, Bruhwiler L, Chatterjee A, Fisher JB, Hashemi J, Parmentier FW, Rogers BM, Westermann S, Watts JD, Blanc‐Betes E, Fuchs M, Kruse S, Malhotra A, Miner K, Strauss J, Armstrong A, Epstein HE, Gay B, Goeckede M, Kalhori A, Kou D, Miller CE, Natali SM, Oh Y, Shakil S, Sonnentag O, Varner RK, Zolkos S, Schuur EAG, Hugelius G (2024) Permafrost Carbon: Progress on Understanding Stocks and Fluxes Across Northern Terrestrial Ecosystems. J Geophys Res Biogeosciences 129:e2023JG007638. https://doi.org/10.1029/2023JG007638

189. Yang S, Wen X, Wu T, Wu X, Wang X, Jin X, Li X, Yang X, Yang L, Wang H (2024) Carbon‐cycling microorganisms in permafrost and their responses to a warming climate: A review. Permafr Periglac Process 35:218–231. https://doi.org/10.1002/ppp.2206

190. Schädel C, Rogers BM, Lawrence DM, Koven CD, Brovkin V, Burke EJ, Genet H, Huntzinger DN, Jafarov E, McGuire AD, Riley WJ, Natali SM (2024) Earth system models must include permafrost carbon processes. Nat Clim Change 14:114–116. https://doi.org/10.1038/s41558-023-01909-9

191. García-Palacios P, Bradford MA, Benavente-Ferraces I, De Celis M, Delgado-Baquerizo M, García-Gil JC, Gaitán JJ, Goñi-Urtiaga A, Mueller CW, Panettieri M, Rey A, Sáez-Sandino T, Schuur EAG, Sokol NW, Tedersoo L, Plaza C (2024) Dominance of particulate organic carbon in top mineral soils in cold regions. Nat Geosci. https://doi.org/10.1038/s41561-023-01354-5

192. Valman S, Siewert MB, Boyd D, Ledger M, Gee D, De La Barreda-Bautista B, Sowter A, Sjögersten S (2024) InSAR-measured permafrost degradation of palsa peatlands in northern Sweden. The Cryosphere 18:1773–1790. https://doi.org/10.5194/tc-18-1773-2024

193. Gao T, Kang S, Yao T, Zhao Y, Shang X, Nie Y, Chen R, Semiletov I, Zhang T, Luo X, Wei D, Zhang Y (2024) Carbon dynamics shift in changing cryosphere and hydrosphere of the Third Pole. Earth-Sci Rev 250:104717. https://doi.org/10.1016/j.earscirev.2024.104717

194. Jiao Y, Zhang Y, Wang X, Altshuler I, Zhou F, Fang M, Rinnan R, Chen J, Wang Z (2024) Awakening: Potential Release of Dormant Chemicals from Thawing Permafrost Soils under Climate Change. Environ Sci Technol acs.est.4c06014. https://doi.org/10.1021/acs.est.4c06014

195. Makopoulou E, Karjalainen O, Elia L, Blais‐Stevens A, Lantz T, Lipovsky P, Lombardo L, Nicu IC, Rubensdotter L, Rudy ACA, Hjort J (2024) Retrogressive thaw slump susceptibility in the northern hemisphere permafrost region. Earth Surf Process Landf esp.5890. https://doi.org/10.1002/esp.5890

196. Miner K, Baskaran L, Gay B, Sousa D, Miller C (2024) Frozen no more, a case study of Arctic permafrost impacts of oil and gas withdrawal. Sci Rep 14:25403. https://doi.org/10.1038/s41598-024-76292-2

197. Mu M, Mu C, Liu H, Zhang C, Jia Y, Lei P, Peng X (2024) Decline of CO_2_ Release During the Evolution of the Thaw Slump on the Northern Qinghai‐Tibet Plateau. J Geophys Res Biogeosciences 129:e2024JG008162. https://doi.org/10.1029/2024JG008162

198. Sabino M, Gustafsson Ö, Wild B, Semiletov IP, Dudarev OV, Ingrosso G, Tesi T (2024) Feedbacks From Young Permafrost Carbon Remobilization to the Deglacial Methane Rise. Glob Biogeochem Cycles 38:e2024GB008164. https://doi.org/10.1029/2024GB008164

199. Strauss J, Marushchak ME, Van Delden L, Sanders T, Biasi C, Voigt C, Jongejans LL, Treat C (2024) Potential nitrogen mobilisation from the Yedoma permafrost domain. Environ Res Lett 19:043002. https://doi.org/10.1088/1748-9326/ad3167

200. Strauss J, Fuchs M, Hugelius G, Miesner F, Nitze I, Opfergelt S, Schuur E, Treat C, Turetsky M, Yang Y, Grosse G (2024) Organic matter storage and vulnerability in the permafrost domain. In: Encyclopedia of Quaternary Science. Elsevier, pp 399–410

201. Hugelius G, Ramage J, Burke E, Chatterjee A, Smallman TL, Aalto T, Bastos A, Biasi C, Canadell JG, Chandra N, Chevallier F, Ciais P, Chang J, Feng L, Jones MW, Kleinen T, Kuhn M, Lauerwald R, Liu J, López‐Blanco E, Luijkx IT, Marushchak ME, Natali SM, Niwa Y, Olefeldt D, Palmer PI, Patra PK, Peters W, Potter S, Poulter B, Rogers BM, Riley WJ, Saunois M, Schuur EAG, Thompson RL, Treat C, Tsuruta A, Turetsky MR, Virkkala A ‐M., Voigt C, Watts J, Zhu Q, Zheng B (2024) Permafrost Region Greenhouse Gas Budgets Suggest a Weak CO_2_ Sink and CH_4_ and N_2_ O Sources, But Magnitudes Differ Between Top‐Down and Bottom‐Up Methods. Glob Biogeochem Cycles 38:e2023GB007969. https://doi.org/10.1029/2023GB007969

202. Rodenhizer H, Yang Y, Fiske G, Potter S, Windholz T, Mullen A, Watts JD, Rogers BM (2024) A Comparison of Satellite Imagery Sources for Automated Detection of Retrogressive Thaw Slumps. Remote Sens 16:2361. https://doi.org/10.3390/rs16132361

203. Yang S, Wen X, Wagner D, Strauss J, Kallmeyer J, Anthony SE, Liebner S (2024) Microbial assemblages in Arctic coastal thermokarst lakes and lagoons. FEMS Microbiol Ecol 100:fiae014. https://doi.org/10.1093/femsec/fiae014

204. Thomas M, Jongejans LL, Strauss J, Vermylen C, Calcus S, Opel T, Kizyakov A, Wetterich S, Grosse G, Opfergelt S (2024) A Third of Organic Carbon Is Mineral Bound in Permafrost Sediments Exposed by the World’s Largest Thaw Slump, Batagay, Siberia. Permafr Periglac Process 35:278–293. https://doi.org/10.1002/ppp.2230

205. Carneiro Barreto MS, Wani RP, Goranov AI, Sowers TD, Fischel M, Douglas TA, Hatcher PG, Sparks DL (2024) Carbon Fate, Iron Dissolution, and Molecular Characterization of Dissolved Organic Matter in Thawed Yedoma Permafrost under Varying Redox Conditions. Environ Sci Technol acs.est.3c08219. https://doi.org/10.1021/acs.est.3c08219

206. Liljedahl AK, Witharana C, Manos E (2024) The capillaries of the Arctic tundra. Nat Water 2:611–614. https://doi.org/10.1038/s44221-024-00276-9

207. Fouché J, Hirst C, Bonneville S, Opfergelt S, Haghipour N, Eglinton TI, Vonk JE, Bröder L (2024) Rainfall Impacts Dissolved Organic Matter and Cation Export From Permafrost Catchments and a Glacial River During Late Summer in Northeast Greenland. Permafr Periglac Process ppp.2250. https://doi.org/10.1002/ppp.2250

208. Ackermann M, Amann C, Cook B, Davies P, Glen A, Frederick J, Holdren J, Ivey M, Leland R, Maddox M (2024) Pan-Arctic Methane

209. Khattak AJ, Hamm A (2024) Limited control of microtopography evolution on ground subsidence in polygonal tundra landscapes. Sci Total Environ 948:174741. https://doi.org/10.1016/j.scitotenv.2024.174741

210. Walter Anthony KM, Anthony P, Hasson N, Edgar C, Sivan O, Eliani-Russak E, Bergman O, Minsley BJ, James SR, Pastick NJ, Kholodov A, Zimov S, Euskirchen E, Bret-Harte MS, Grosse G, Langer M, Nitzbon J (2024) Upland Yedoma taliks are an unpredicted source of atmospheric methane. Nat Commun 15:6056. https://doi.org/10.1038/s41467-024-50346-5

211. Lenton TM, Abrams JF, Bartsch A, Bathiany S, Boulton CA, Buxton JE, Conversi A, Cunliffe AM, Hebden S, Lavergne T, Poulter B, Shepherd A, Smith T, Swingedouw D, Winkelmann R, Boers N (2024) Remotely sensing potential climate change tipping points across scales. Nat Commun 15:343. https://doi.org/10.1038/s41467-023-44609-w

212. Smith MI, Ke Y, Geyman EC, Reahl JN, Douglas MM, Seelen EA, Magyar JS, Dunne KBJ, Mutter EA, Fischer WW, Lamb MP, West AJ (2024) Mercury stocks in discontinuous permafrost and their mobilization by river migration in the Yukon River Basin. Environ Res Lett 19:084041. https://doi.org/10.1088/1748-9326/ad536e

213. Huang Y, Huang L, Qiu C, Ciais P (2024) Evaluation of effects of heat released from SOC decomposition on soil carbon stock and temperature. Glob Change Biol 30:e17391. https://doi.org/10.1111/gcb.17391

214. Zhou G, Liu W, Xie C, Song X, Zhang Q, Li Q, Liu G, Li Q, Luo B (2024) Accelerating thermokarst lake changes on the Qinghai–Tibetan Plateau. Sci Rep 14:2985. https://doi.org/10.1038/s41598-024-52558-7

215. Parmentier F-JW, Thornton BF, Silyakova A, Christensen TR (2024) Vulnerability of Arctic-Boreal methane emissions to climate change. Front Environ Sci 12:1460155. https://doi.org/10.3389/fenvs.2024.1460155

216. Abernethy S, Jackson RB (2024) Atmospheric methane removal may reduce climate risks. Environ Res Lett 19:051001. https://doi.org/10.1088/1748-9326/ad3b22

217. Jenrich M, Angelopoulos M, Liebner S, Treat C, Knoblauch C, Yang S, Grosse G, Giebeler F, Jongejans LL, Grigoriev M, Strauss J (2024) Greenhouse Gas Production and Microbial Response During the Transition From Terrestrial Permafrost to a Marine Environment. Permafr Periglac Process ppp.2251. https://doi.org/10.1002/ppp.2251

218. Keskitalo KH, Bröder L, Tesi T, Mann PJ, Jong DJ, Bulte Garcia S, Davydova A, Davydov S, Zimov N, Haghipour N, Eglinton TI, Vonk JE (2024) Seasonal particulate organic carbon dynamics of the Kolyma River tributaries, Siberia. Biogeosciences 21:357–379. https://doi.org/10.5194/bg-21-357-2024

219. Liu Z, Rogers BM, Keppel-Aleks G, Helbig M, Ballantyne AP, Kimball JS, Chatterjee A, Foster A, Kaushik A, Virkkala A-M, Burrell AL, Schwalm C, Sweeney C, Schuur EAG, Dean J, Watts JD, Kim JE, Wang JA, Hu L, Welp L, Berner LT, Mauritz M, Mack M, Parazoo NC, Madani N, Keeling R, Commane R, Goetz S, Piao S, Natali SM, Wang W, Buermann W, Walker X, Lin X, Wang X, Jin Y, Yu K, Zhang Y (2024) Seasonal CO2 amplitude in northern high latitudes. Nat Rev Earth Environ 5:802–817. https://doi.org/10.1038/s43017-024-00600-7

220. Bartsch A, Efimova A, Widhalm B, Muri X, Von Baeckmann C, Bergstedt H, Ermokhina K, Hugelius G, Heim B, Leibman M (2024) Circumarctic land cover diversity considering wetness gradients. Hydrol Earth Syst Sci 28:2421–2481. https://doi.org/10.5194/hess-28-2421-2024

221. Anderson S, Cochran C, Anderson R, Repasch M, Arcuri J, Overeem I (2024) A conceptual carbon budget for an icy riverine corridor. International Permafrost Association (IPA)

222. Mu C, Song J, Liu H, Peng X, Zheng L, Gao Z, Sun H, Fan C, Mu M, Guo L (2024) Impacts of increasing land-ocean interactions on carbon cycles in the Arctic. Earth Crit Zone 1:100010. https://doi.org/10.1016/j.ecz.2024.100010

223. Crumley RL, Bachand CL, Bennett KE (2024) Snow Distribution Patterns Revisited: A Physics‐Based and Machine Learning Hybrid Approach to Snow Distribution Mapping in the Sub‐Arctic. Water Resour Res 60:e2023WR036180. https://doi.org/10.1029/2023WR036180

224. Gorham KA, Abernethy S, Jones TR, Hess P, Mahowald NM, Meidan D, Johnson MS, Van Herpen MMJW, Xu Y, Saiz-Lopez A, Röckmann T, Brashear CA, Reinhardt E, Mann D (2024) Opinion: A research roadmap for exploring atmospheric methane removal via iron salt aerosol. Atmospheric Chem Phys 24:5659–5670. https://doi.org/10.5194/acp-24-5659-2024

225. Seiler C, Kou‐Giesbrecht S, Arora VK, Melton JR (2024) The Impact of Climate Forcing Biases and the Nitrogen Cycle on Land Carbon Balance Projections. J Adv Model Earth Syst 16:e2023MS003749. https://doi.org/10.1029/2023MS003749

226. Schimel DS, Carroll D (2024) Carbon Cycle–Climate Feedbacks in the Post-Paris World. Annu Rev Earth Planet Sci 52:467–493. https://doi.org/10.1146/annurev-earth-031621-081700

227. Turner MG, Calder WJ, Cumming GS, Hughes TP, Jentsch A, LaDeau SL, Lenton TM, Shuman BN, Turetsky MR, Ratajczak Z, Williams JW, Williams AP, Carpenter SR (2020) Climate change, ecosystems and abrupt change: science priorities. Philos Trans R Soc B Biol Sci 375:20190105. https://doi.org/10.1098/rstb.2019.0105

228. Burn CR, Friele PA (1989) Geomorphology, Vegetation Succession, Soil Characteristics and Permafrost in Retrogressive Thaw Slumps near Mayo, Yukon Territory. Arctic 42:31–40

229. Lantz TC, Kokelj SV, Gergel SE, Henry GHR (2009) Relative impacts of disturbance and temperature: persistent changes in microenvironment and vegetation in retrogressive thaw slumps. Glob Change Biol 15:1664–1675. https://doi.org/10.1111/j.1365-2486.2009.01917.x

230. Lewkowicz AG (2007) Dynamics of active‐layer detachment failures, Fosheim Peninsula, Ellesmere Island, Nunavut, Canada. Permafr Periglac Process 18:89–103. https://doi.org/10.1002/ppp.578

231. Irrgang AM, Bendixen M, Farquharson LM, Baranskaya AV, Erikson LH, Gibbs AE, Ogorodov SA, Overduin PP, Lantuit H, Grigoriev MN, Jones BM (2022) Drivers, dynamics and impacts of changing Arctic coasts. Nat Rev Earth Environ 3:39–54. https://doi.org/10.1038/s43017-021-00232-1

232. Nielsen DM, Pieper P, Barkhordarian A, Overduin P, Ilyina T, Brovkin V, Baehr J, Dobrynin M (2022) Increase in Arctic coastal erosion and its sensitivity to warming in the twenty-first century. Nat Clim Change 12:263–270. https://doi.org/10.1038/s41558-022-01281-0

233. Mackay JR, Burn CR (2011) A Century (1910–2008) of Change in a Collapsing Pingo, Parry Peninsula, Western Arctic Coast, Canada. Permafr Periglac Process 22:266–272. https://doi.org/10.1002/ppp.723

234. Mackay JR (2002) Pingo Growth and collapse, Tuktoyaktuk Peninsula Area, Western Arctic Coast, Canada: a long-term field study. Géographie Phys Quat 52:271–323. https://doi.org/10.7202/004847ar

235. Chuvilin EM, Sokolova NS, Bukhanov BA, Davletshina DA, Spasennykh MY (2021) Formation of Gas-Emission Craters in Northern West Siberia: Shallow Controls. Geosciences 11:393. https://doi.org/10.3390/geosciences11090393

236. Zolkos S, Fiske G, Windholz T, Duran G, Yang Z, Olenchenko V, Faguet A, Natali SM (2021) Detecting and Mapping Gas Emission Craters on the Yamal and Gydan Peninsulas, Western Siberia. Geosciences 11:21. https://doi.org/10.3390/geosciences11010021

237. Benedict JB (1976) Frost Creep and Gelifluction Features: A Review. Quat Res 6:55–76. https://doi.org/10.1016/0033-5894(76)90040-5

238. Jorgenson T, Harden J, Kanevskiy M, O’Donnell J, Wickland K, Ewing S, Manies K, Zhuang Q, Shur Y, Striegl R, Koch J (2013) Reorganization of vegetation, hydrology and soil carbon after permafrost degradation across heterogeneous boreal landscapes. Environ Res Lett 8:035017. https://doi.org/10.1088/1748-9326/8/3/035017

239. Kokelj SV, Jorgenson MT (2013) Advances in Thermokarst Research. Permafr Periglac Process 24:108–119. https://doi.org/10.1002/ppp.1779

240. Fuchs M, Nitze I, Strauss J, Günther F, Wetterich S, Kizyakov A, Fritz M, Opel T, Grigoriev MN, Maksimov GT, Grosse G (2020) Rapid Fluvio-Thermal Erosion of a Yedoma Permafrost Cliff in the Lena River Delta. Front Earth Sci 8:336. https://doi.org/10.3389/feart.2020.00336

241. Rowland JC, Schwenk JP, Shelef E, Muss J, Ahrens D, Stauffer S, Pilliouras A, Crosby B, Chadwick A, Douglas MM, Kemeny PC, Lamb MP, Li GK, Vulis L (2023) Scale‐Dependent Influence of Permafrost on Riverbank Erosion Rates. J Geophys Res Earth Surf 128:e2023JF007101. https://doi.org/10.1029/2023JF007101

242. Walker J, Arnborg L, Peippo J (1987) Riverbank Erosion in the Colville Delta, Alaska. Geogr Ann Ser Phys Geogr 69:61–70. https://doi.org/10.1080/04353676.1987.11880197

243. Farquharson LM, Romanovsky VE, Kholodov A, Nicolsky D (2022) Sub-aerial talik formation observed across the discontinuous permafrost zone of Alaska. Nat Geosci 15:475–481. https://doi.org/10.1038/s41561-022-00952-z

244. O’Neill HB, Roy‐Leveillee P, Lebedeva L, Ling F (2020) Recent advances (2010–2019) in the study of taliks. Permafr Periglac Process 31:346–357. https://doi.org/10.1002/ppp.2050

245. O’Neill HB, Burn CR (2017) Talik Formation at a Snow Fence in Continuous Permafrost, Western Arctic Canada. Permafr Periglac Process 28:558–565. https://doi.org/10.1002/ppp.1905

246. Devoie É, Connon RF, Beddoe R, Goordial J, Quinton WL, Craig JR (2024) Disconnected active layers and unfrozen permafrost: A discussion of permafrost-related terms and definitions. Sci Total Environ 912:169017. https://doi.org/10.1016/j.scitotenv.2023.169017

247. Bowden WB, Gooseff MN, Balser A, Green A, Peterson BJ, Bradford J (2008) Sediment and nutrient delivery from thermokarst features in the foothills of the North Slope, Alaska: Potential impacts on headwater stream ecosystems. J Geophys Res Biogeosciences 113:2007JG000470. https://doi.org/10.1029/2007JG000470

248. Fortier D, Allard M, Shur Y (2007) Observation of rapid drainage system development by thermal erosion of ice wedges on Bylot Island, Canadian Arctic Archipelago. Permafr Periglac Process 18:229–243. https://doi.org/10.1002/ppp.595

249. Godin E, Osinski GR, Harrison TN, Pontefract A, Zanetti M (2019) Geomorphology of Gullies at Thomas Lee Inlet, Devon Island, Canadian High Arctic. Permafr Periglac Process 30:19–34. https://doi.org/10.1002/ppp.1992

250. Godin E, Fortier D (2012) Geomorphology of a thermo-erosion gully, Bylot Island, Nunavut, Canada. Can J Earth Sci 49:979–986. https://doi.org/10.1139/e2012-015

251. Grosse G, Jones B, Arp C (2013) 8.21 Thermokarst Lakes, Drainage, and Drained Basins. In: Treatise on Geomorphology. Elsevier, pp 325–353

252. Osterkamp TE, Viereck L, Shur Y, Jorgenson MT, Racine C, Doyle A, Boone RD (2000) Observations of Thermokarst and Its Impact on Boreal Forests in Alaska, U.S.A. Arct Antarct Alp Res 32:303–315. https://doi.org/10.1080/15230430.2000.12003368

253. Jorgenson MT, Kanevskiy MZ, Jorgenson JC, Liljedahl A, Shur Y, Epstein H, Kent K, Griffin CG, Daanen R, Boldenow M, Orndahl K, Witharana C, Jones BM (2022) Rapid transformation of tundra ecosystems from ice-wedge degradation. Glob Planet Change 216:103921. https://doi.org/10.1016/j.gloplacha.2022.103921

254. McNamara JP, Kane DL, Hinzman LD (1998) An analysis of streamflow hydrology in the Kuparuk River Basin, Arctic Alaska: a nested watershed approach. J Hydrol 206:39–57. https://doi.org/10.1016/S0022-1694(98)00083-3

255. Trochim ED, Prakash A, Kane DL, Romanovsky VE (2016) Remote sensing of water tracks. Earth Space Sci 3:106–122. https://doi.org/10.1002/2015EA000112

256. Gibson CM, Chasmer LE, Thompson DK, Quinton WL, Flannigan MD, Olefeldt D (2018) Wildfire as a major driver of recent permafrost thaw in boreal peatlands. Nat Commun 9:3041. https://doi.org/10.1038/s41467-018-05457-1

257. Baltzer JL, Veness T, Chasmer LE, Sniderhan AE, Quinton WL (2014) Forests on thawing permafrost: fragmentation, edge effects, and net forest loss. Glob Change Biol 20:824–834. https://doi.org/10.1111/gcb.12349

258. Camill P (2005) Permafrost Thaw Accelerates in Boreal Peatlands During Late-20th Century Climate Warming. Clim Change 68:135–152. https://doi.org/10.1007/s10584-005-4785-y

259. Carpino OA, Berg AA, Quinton WL, Adams JR (2018) Climate change and permafrost thaw-induced boreal forest loss in northwestern Canada. Environ Res Lett 13:084018. https://doi.org/10.1088/1748-9326/aad74e

260. Helbig M, Pappas C, Sonnentag O (2016) Permafrost thaw and wildfire: Equally important drivers of boreal tree cover changes in the Taiga Plains, Canada. Geophys Res Lett 43:1598–1606. https://doi.org/10.1002/2015GL067193

261. Jafarov EE, Romanovsky VE, Genet H, McGuire AD, Marchenko SS (2013) The effects of fire on the thermal stability of permafrost in lowland and upland black spruce forests of interior Alaska in a changing climate. Environ Res Lett 8:035030. https://doi.org/10.1088/1748-9326/8/3/035030

262. Jones BM, Baughman CA, Romanovsky VE, Parsekian AD, Babcock EL, Stephani E, Jones MC, Grosse G, Berg EE (2016) Presence of rapidly degrading permafrost plateaus in south-central Alaska. The Cryosphere 10:2673–2692. https://doi.org/10.5194/tc-10-2673-2016

263. Lara MJ, Genet H, McGuire AD, Euskirchen ES, Zhang Y, Brown DRN, Jorgenson MT, Romanovsky V, Breen A, Bolton WR (2016) Thermokarst rates intensify due to climate change and forest fragmentation in an Alaskan boreal forest lowland. Glob Change Biol 22:816–829. https://doi.org/10.1111/gcb.13124

264. Frost GV, Loehman RA, Saperstein LB, Macander MJ, Nelson PR, Paradis DP, Natali SM (2020) Multi-decadal patterns of vegetation succession after tundra fire on the Yukon-Kuskokwim Delta, Alaska. Environ Res Lett 15:025003. https://doi.org/10.1088/1748-9326/ab5f49

265. Alexander HD, Natali SM, Loranty MM, Ludwig SM, Spektor VV, Davydov S, Zimov N, Trujillo I, Mack MC (2018) Impacts of increased soil burn severity on larch forest regeneration on permafrost soils of far northeastern Siberia. For Ecol Manag 417:144–153. https://doi.org/10.1016/j.foreco.2018.03.008

266. Barrett K, Rocha AV, Van De Weg MJ, Shaver G (2012) Vegetation shifts observed in arctic tundra 17 years after fire. Remote Sens Lett 3:729–736. https://doi.org/10.1080/2150704X.2012.676741

267. Gaglioti BV, Berner LT, Jones BM, Orndahl KM, Williams AP, Andreu‐Hayles L, D’Arrigo RD, Goetz SJ, Mann DH (2021) Tussocks Enduring or Shrubs Greening: Alternate Responses to Changing Fire Regimes in the Noatak River Valley, Alaska. J Geophys Res Biogeosciences 126:e2020JG006009. https://doi.org/10.1029/2020JG006009

268. Holloway JE, Lewkowicz AG, Douglas TA, Li X, Turetsky MR, Baltzer JL, Jin H (2020) Impact of wildfire on permafrost landscapes: A review of recent advances and future prospects. Permafr Periglac Process 31:371–382. https://doi.org/10.1002/ppp.2048

269. Li X-Y, Jin H-J, Wang H-W, Marchenko SS, Shan W, Luo D-L, He R-X, Spektor V, Huang Y-D, Li X-Y, Jia N (2021) Influences of forest fires on the permafrost environment: A review. Adv Clim Change Res 12:48–65. https://doi.org/10.1016/j.accre.2021.01.001

270. Rocha AV, Loranty MM, Higuera PE, Mack MC, Hu FS, Jones BM, Breen AL, Rastetter EB, Goetz SJ, Shaver GR (2012) The footprint of Alaskan tundra fires during the past half-century: implications for surface properties and radiative forcing. Environ Res Lett 7:044039. https://doi.org/10.1088/1748-9326/7/4/044039
